# Extensive genetic diversity of severe fever with thrombocytopenia syndrome virus circulating in Hubei Province, China, 2018–2022

**DOI:** 10.1371/journal.pntd.0011654

**Published:** 2023-09-18

**Authors:** Yu-ting Ren, Hong-pan Tian, Jia-le Xu, Man-qing Liu, Kun Cai, Shu-liang Chen, Xue-bing Ni, Yi-rong Li, Wei Hou, Liang-jun Chen

**Affiliations:** 1 State Key Laboratory of Virology/Department of Laboratory Medicine/Hubei Provincial Key Laboratory of Allergy and Immunology, Zhongnan Hospital/School of Basic Medical Sciences, Wuhan University, Wuhan, China; 2 Division of Virology, Wuhan Center for Disease Control & Prevention, Wuhan, China; 3 Institute of Health Inspection and Testing, Hubei Provincial Center for Disease Control & Prevention, Wuhan, China; 4 State Key Laboratory of Emerging Infectious Diseases and Centre of Influenza Research, School of Public Health, The University of Hong Kong, Hong Kong SAR, P. R. China; 5 School of Public Health, Wuhan University, Wuhan, China; Temple University, UNITED STATES

## Abstract

Severe fever with thrombocytopenia syndrome virus (SFTSV), an etiological agent causing febrile human disease was identified as an emerging tick-borne bunyavirus. The clinical disease characteristics and case fatality rates of SFTSV may vary across distinct regions and among different variant genotypes. From 2018 to 2022, we surveyed and recruited 202 severe fever with thrombocytopenia syndrome (SFTS) patients in Hubei Province, a high-incidence area of the epidemic, and conducted timely and systematic research on the disease characteristics, SFTSV diversity, and the correlation between virus genome variation and clinical diseases. Our study identified at least 6 genotypes of SFTSV prevalent in Hubei Province based on the analysis of the S, M, and L genome sequences of 88 virus strains. Strikingly, the dominant genotype of SFTSV was found to change during the years, indicating a dynamic shift in viral genetic diversity in the region. Phylogenetic analysis revealed the genetic exchange of Hubei SFTSV strains was relatively frequent, including 3 reassortment strains and 8 recombination strains. Despite the limited sample size, SFTSV C1 genotype may be associated with higher mortality compared to the other four genotypes, and the serum amyloid A (SAA) level, an inflammatory biomarker, was significantly elevated in these patients. Overall, our data summarize the disease characteristics of SFTSV in Hubei Province, highlight the profound changes in viral genetic diversity, and indicate the need for in-depth monitoring and exploration of the relationship between viral mutations and disease severity.

## Author summary

Severe fever with thrombocytopenia syndrome virus (SFTSV), an emerging tick-borne bunyavirus, caused febrile human disease. Studies have shown that the virus was constantly mutating, and the clinical features of distinct mutational genotypes were also different. Therefore, routine surveillance of this disease’s epidemic features is necessary, especially in high-incidence areas. In this study, we conducted timely and systematic research on the disease characteristics, SFTSV diversity, and the correlation between virus genome variation and clinical diseases based on 202 severe fevers with thrombocytopenia syndrome (SFTS) patients in Hubei Province, from 2018 to 2022. Our findings identified at least six prevalent genotypes of SFTSV in Hubei Province and revealed that the dominant genotype of the virus changed over time, indicating a complex genotype diversity of Hubei SFTSV strains. Frequent genetic exchange was also revealed by phylogenetic analysis involving 3 reassortment strains and 8 recombination strains. Despite the limited sample size, SFTSV C1 genotype may be relevant to higher mortality with significant augment level of inflammatory marker serum amyloid A (SAA) than other four genotypes. This study updated the disease characteristics of SFTS with SFTSV epidemic diversity in Hubei Province and provided important insights into the relationship between viral mutations and disease severity.

## Introduction

Severe fever with thrombocytopenia syndrome (SFTS) is an emerging tick-borne hemorrhagic fever, which initially appeared in the rural areas of Hubei and Henan Provinces, central China, with clinical manifestations including fever, thrombocytopenia, leukocytopenia and regional lymphadenopathy or even multiorgan failure observed in severe patients [1]. Since 2010, SFTS cases have been subsequently reported in Japan [2] and South Korea [3], similar virus like Heartland virus infected cases were also reported in the USA [4,5]. Tick bite has been identified to be the main transmitted route of SFTSV and *Haemaphysalis longicornis* is a competent vector to transmit this virus[6]. Moreover, previous studies found that direct contact with the blood or bloody secretions of SFTS patients can also lead to SFTSV infection, suggesting the possibility of human-to-human transmission of the virus [7]. Due to the high expanding epidemic and high lethality with the lack of specific drug treatment and vaccine prevention, the disease remains a huge threat to public health and safety.

The etiological agent of this disease is SFTSV, a novel *Bandavirus* of family *Phenuiviridae*, recently named *Dabie Bandavirus* by ICTV [8]. Like other phleboviruses, the genome of SFTSV is composed of three single-stranded negative RNA fragments, including L, M and S segments [1]. Up to 2017, at least 10 genotypes of SFTSV were prevalent in East Asia, including the C1-C5 genotype defined as the Chinese lineage, the J1-J3 genotype defined as the Japanese lineage [9]. With the enrichment of SFTSV sequences, crossover events between different genetic lineages of SFTSV have been found, reflecting the potential for overseas transmission of SFTSV by migratory birds [10]. Based on the fragment nature of SFTSV, the evolutionary characteristics showed high substitution rate with recombination and reassorted strains (9.3%, 8.0% and 7.4% for S, M, and L segments, respectively), resulting in the geographically extensive co-circulation in virus strains [11,12]. In comparison with other viral member in the same *Phenuiviridae* family, such as Rift Valley fever virus (RVFV), SFTSV characterized as more variable [13]. Therefore, the epidemic characteristics of SFTSV show frequent variation and cross-regional transmission worldwide.

The case fatality rate (CFR) of SFTS showed distinct locational differentiation, ranging from 5.3% to 16.2% in China, and 27% in Japan, and 23.3% in South Korea [14]. In 2019, Yun et al. [15] analyzed the full-length sequence of SFTSVs isolated from serum of 116 SFTS patients in South Korea, and found genotype B exhibited the highest CFR in South Korea, suggesting that SFTSV pathogenicity may be genotype-dependent. Similarly, an analysis of more than 800 SFTSV strains in Xinyang, Henan Province in 2021 found that one specific clade (IV) was significantly associated with increased CFR in Henan SFTSV strain, indicating the impact of viral genomic variations on SFTS mortality [16]. These results demonstrated that the genetic evolution of SFTSV strains in different regions might be related to the clinical outcome of the patients. Hubei Province is one of the most epidemic areas of SFTSV, where initially all SFTSV strains originally belonged to the genotype C3 [10]. However, subsequent studies found that the C2 and C3 genotypes were co-prevalent in Hubei Province [17]. These findings illustrate that SFTSVs circulating in Hubei Province are undergoing dynamic evolution with constant genetic variation, and multiple genotypes are co-circulating. Therefore, it is necessary to continuously monitor the epidemiological variation of SFTSV in Hubei Province, especially to identify and analyze the clinical characteristics of different variants.

In this study, serum samples with corresponding epidemiological information were collected from 202 confirmed SFTS patients in Hubei Province from 2018 to 2022. Due to variations in the time of serum collection post-onset, SFTSV RNA were detected in 88 confirmed SFTS patients and 45 viral full-length genomic sequences were obtained. This study investigated the clinical characteristics of SFTS patients and analyzed the genetic evolution features of SFTSV in Hubei Province. Furthermore, the relationship between the genetic variation of SFTSV and the disease severity of SFTS patients were preliminarily explored.

## Materials and methods

### Ethics statement

All samples were collected as part of public health diagnostic activities for SFTS, were pre-existing relative to the start of the study, and were examined as anonymous samples. In accordance with the medical research regulations in China, the research scheme was authorized by the medical Ethics Committee of Zhongnan Hospital of Wuhan University (No. 2019125).

### Clinical samples and participants

A total of 202 patients who were hospitalized in Zhongnan Hospital of Wuhan University and clinically diagnosed as SFTS from May 2018 to July 2022 were enrolled in our study. Patients were included in this study if they had been diagnosed as SFTS, defined as meeting at least one of the diagnostic criteria stipulated by the Chinese Ministry of Health [18]. Patients’ samples and information were analyzed in this study with providing written informed consent from all participants or their guardians. Patients who experienced adverse clinical progression or financial difficulty and discontinued therapy or had been discharged from hospital were followed up by phone calls or home visits within two weeks to determine their final outcome (fatal or survival). All the serum samples and laboratory variables (including SAA) were collected in the disease progression period.

### RNA extraction, PCR, sequencing and phylogenetic analysis

202 serum samples from these SFTS patients were collected and viral RNA was extracted using a Nucleic Acid Isolation and Purification Kit (DAAN GENE, Guangzhou, China). SFTSV RNA was detected by nested RT-PCR, which consisted of two rounds of PCR with two pairs of primers based on L segment set to identify nucleic acid positive samples (LF [5’-GTYCAGTGGTCYCTYTGGGT-3’], LF’[5’-GAARTT CTGGCCHATCTAYGTYATYATC-3’], LR [3’-GYTCTGCYHCCAHTGYCTYCC-5’] and LR’ [3’-CCAAT GTACCAGCTRTTYACCAT-5’]) [19]. The first round of amplification for SFTSV RNA detection used the PrimeScript One Step RT–PCR Kit (Takara, Dalian, China), which involved the following conditions: an initial step of 30 min at 50°C for reverse transcription and 3 min at 95°C for denaturation, followed by 36 cycles of 35 s at 94°C, 35 s at 54°C, and 45 s at 72°C, and a final extension step of 5 min at 72°C. The second round of amplification was performed using the 2×Taq PCR MasterMix II (TIANGEN, Beijing, China) with an initial step of 3 min at 94°C for denaturation, followed by 36 cycles of 35 s at 94°C, 35 s at 54°C, and 90 s at 72°C, and a final extension step of 5 min at 72°C.

To prevent contamination, various measures were taken during the study, such as cleaning the operating floor and pipettes using 75% alcohol and nucleic acid scavenger before and after experiments, replacing reagents regularly, and setting multiple negative controls in every nested RT-PCR. Additionally, to mitigate the potential for mutations in the genomes during RT-PCR, multiple amplicons were generated for sequencing. To gain an initial understanding of the characteristic of genetic variation and evolutionary relationship in SFTSV, specific primers were designed to amplified the partial sections of L, M and S segments of SFTSV. The specific primers used in this study were L1F, L1R, LN-1F and LN-1R; M1F, M1R, MN-1F and MN-1R; S1F, S1R, SN-1F and SN-1R, which were listed in S1 Table. To further explore the characteristic of genetic variation including reassortment and recombination, we selected 45 samples to obtain full-length genome sequences of SFTSV using a total of 14, 10 and 6 pairs of primers for L, M and S segment respectively (S1 Table). The selection of these samples was based on the principle of covering all genotypes and disease severity, and all selected cases had as complete disease course information as possible. After amplification, the obtained sequences were assembled using the SeqMan program in DNAStar to obtain full-length sequences of SFTSV. All the partial and complete sequences of SFTSV obtained in this study have been deposited to GenBank under the accession numbers: OQ388786-OQ388869, OQ388741-OQ388785 (L segment), OQ388871-OQ388954, OQ388955-OQ388999 (M segment) and OQ389000-OQ389080, OQ389081-OQ389125 (S segment), respectively (S2 Table).

Phylogenetic trees were reconstructed based on 313 SFTSV L-segment sequences, 308 SFTSV M-segment sequences and 522 SFTSV S-segment sequences available from GenBank to investigate viral genetic diversity and variation (S3 Table). Model selection using IQ-TREE was performed, and the maximum likelihood method (ML) based on General Time Reversible model combining invariant sites and a gamma distribution (GTR+I+G4) was used to reconstruct the phylogenetic trees with a Nearest-Neighbor-Interchange (NNI) Heuristic method and bootstrap support values calculated from 1000 replicates implemented in MEGA7.0 [20].

### Reassortment and recombination analysis of SFTSV

The method for identifying recombinants was described previously [21,22]. To seek potential recombination events, seven recombination detection methods (RDP, GENECONV, Bootscan, Maxchi, Chimaera, Siscan, and 3Seq) were performed in the aligned sequences using the Recombination Detection Program v4.101 (RDP4) package [23]. To determine the potential recombination events, Similarity plot analysis and Bootscanning plot analysis were conducted by SimPlot software.

The same strains showed inconsistent genotypes determined from L, M and S phylogenetic tree were considered to have potential genetic reassortment event [16]. Full-length of L, M, and S segment sequences of SFTSV were concatenated in order and analyzed with RDP4 package. A breakpoint of recombination detected at the end(s) of segments was defined as a reassortment event [24]. A confirmed recombination event was only considered when the event could be verified by four or more methods with *p*-values<0.05, while avoiding false positive results by Bonferroni correction.

### Statistical analysis

The clinical data were analyzed using the statistical software package SPSS 27.0 (SPSS, an IBM Company, Armonk, NY, USA). Continuous variables were summarized as mean (SD) or median (IQR), and categorical variables were summarized as frequencies and proportions. To compare the clinical parameters and clinical manifestation between dead and survival patients, χ^2^ test, t test, Fisher’s exact test, ANOVA test and nonparametric test were carried out appropriately.

To evaluated the correlation between clinical statistics or clinical manifestation and case fatality, a univariate logistic regression model was used, and the adjusted odds ratio (OR) for each clinical sign or symptom and case severity was calculated using a logistic regression model adjusting for age, sex, and delay from onset to admission.

## Results

### Demographic and clinical characteristics of SFTS patients from Hubei Province, 2018–2022

**Fig 1:**
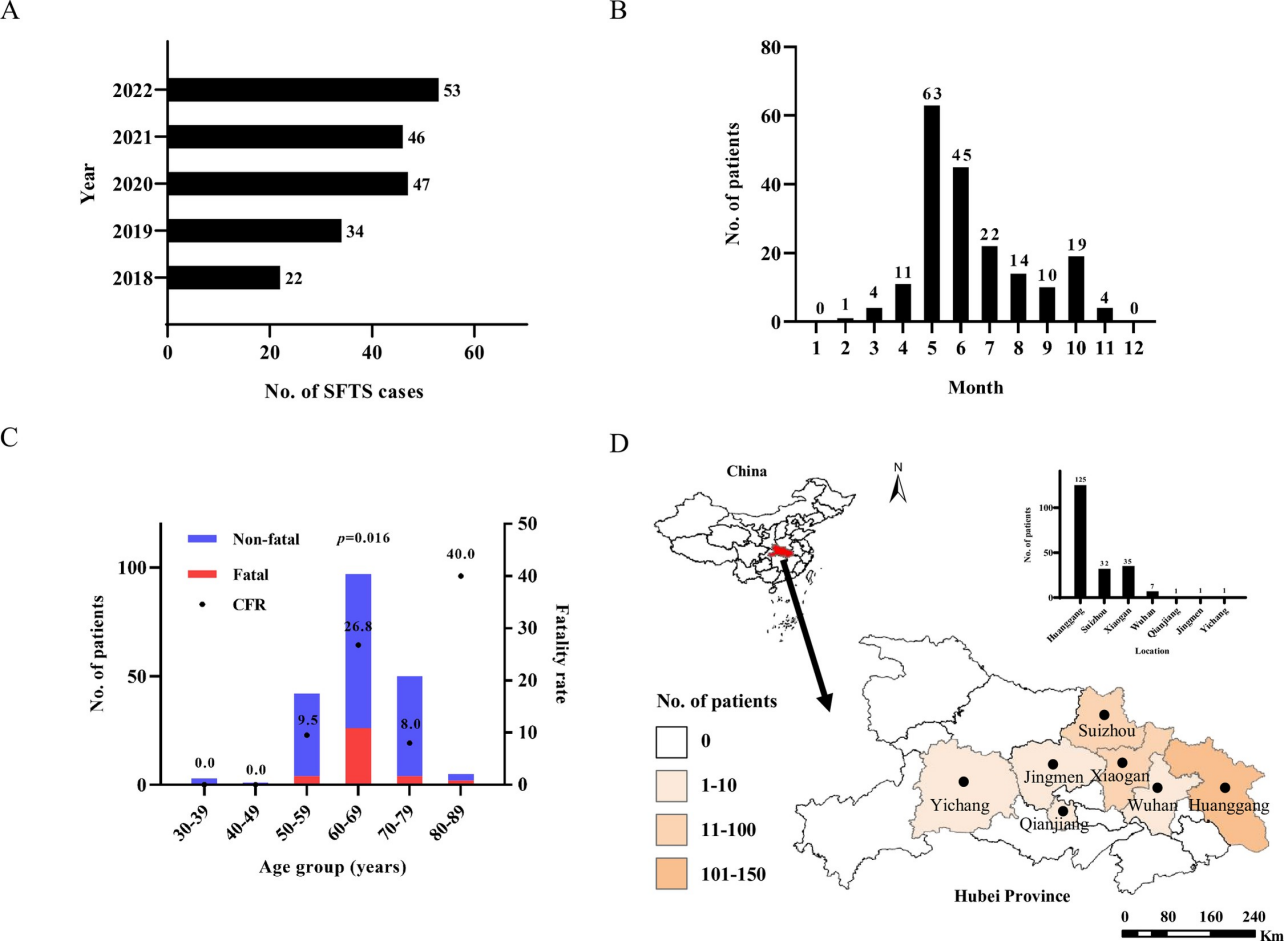
Epidemiological data of SFTS cases in Hubei Province from 2018 to 2022. (A) Confirmed cases of SFTS in Hubei Province from 2018 to 2022. (B) Month distribution of confirmed SFTS cases. (C) Ratio of nonfatal to fatal cases by age group with the case fatality rate (CFR) showed with the datapoints. Blue represented non-fatal cases and red represented fatal cases. (D) Spatial arrangement of confirmed SFTS patients (n = 202) residing in Hubei Province. The map was created in ArcGIS 10.2 software (ESRI Inc., Redlands, CA, USA) and modified using Adobe illustrator, Version CC2018 (Adobe, San Jose, CA, USA). The source of the base layer shapefile was from the open access platform: National Platform for Common Geospatial Information Services (www.tianditu.gov.cn). The black dots indicate the residential regions of confirmed SFTS patients in this study.

A total of 202 patients who were hospitalized in Zhongnan Hospital of Wuhan University and clinically-diagnosed SFTS from May 2018 to July 2022 were recruited in our study (Fig 1A). The confirmed SFTS cases were mostly distributed from March to November (99.5%), and were prevalent from May to July, which was consistent with the peak of tick prevalence (Fig 1B). The age of these patients ranged from 35 to 84 years (64.69±8.31), and the majority cases distributed in the age >50 years. Moreover, the fatality rates were significantly different in SFTS patients in different age groups (*p* = 0.016) (Fig 1C), suggesting that age may be the factor leading to a more serious condition. All of confirmed SFTS patients resided in Hubei Province, with the majority coming from Huanggang, Suizhou and Xiaogan and a minority coming from Wuhan, Jinmen, Qianjiang and Yichang (Fig 1D).

The most common clinical manifestations of these 202 SFTS patients were thrombocytopenia (99.4%), fever (98.7%), leukocytopenia (93.1%), fatigue (68.6%) and diarrhea (61.0%) in the order of the frequency of occurrence (S4 Table). The main clinical manifestations were grouped into non-specific, gastrointestinal, respiratory, hemorrhagic and neurological symptoms (S1 Fig). Among these, neurological symptoms showed high risk for fatal outcome, such as drowsiness (adjusted OR 4.180, 95% CI 1.691–10.332; *p* = 0.002), neural trance (adjusted OR 6.701, 95% CI 2.679–16.762; *p*<0.001) and conscious disturbance (adjusted OR 11.879, 95% CI 4.315–32.703; *p*<0.001) (Table 1). To identify the influencing factors of SFTS fatal outcome and the cause of severe illness, we evaluated the association between each demographic, clinical manifestations, and laboratory variables with SFTS fatal outcome using a logistic regression model adjusting for age, sex, and delay from onset to admission. Indeed, high viral load and 20 laboratory variables were found to present significant effect in fatal SFTS patients. To conclude, our study characterized the demographic and clinical data of 202 SFTS patients from Hubei Province with an adequate sample size and long-time span (2018–2022, 5 years).

**Table 1:** Association of demographic, clinical variables and clinical manifestations with fatal outcome in the SFTS patients.

	Total	Fatal patients	Survival patients	Adjusted OR (95% CI)	*p* value
**Sex [no. (%)]**					
Male	103 (52.0)	21 (58.3)	82 (50.6)	1.366 (0.658–2.836)	0.403
Female	95 (48.0)	15 (41.7)	80 (49.4)		
**Age, y**					
Mean ± SD	64.69±8.31	65.83±7.10	64.44±8.55	1.021 (0.976–1.069)	0.362
**Days since symptom onset**					
Median (IQR)	7 (5–8)	7 (5–7.25)	7 (5–8)	0.971 (0.871–1.083)	0.601
**Underlying disease [no. (%)]**					
Hypertension	51 (26.0)	9 (25.7)	42 (26.1)	1.265 (0.481–3.327)	0.634
Diabetes	17 (8.7)	4 (11.4)	13 (8.1)	1.391 (0.339–5.714)	0.647
Hepatitis	1 (0.5)	0 (0.0)	1 (0.6)		1.000
Heart disease	16 (8.2)	2 (5.7)	14 (8.7)	0.804 (0.162–3.994)	0.790
Cerebrovascular disease	7 (3.6)	3 (8.6)	4 (2.5)	4.424 (0.789–24.810)	0.091
Chronic obstructive pulmonary disease	7 (3.6)	4 (11.4)	3 (1.9)	5.628 (0.954–33.184)	0.056
Gastrointestinal diseases	6 (3.1)	1 (2.9)	5 (3.1)	1.611 (0.159–16.374)	0.687
**Clinical manifestation**					
Multiorgan failure	41 (25.8)	21 (67.7)	20 (15.6)	10.897 (4.350–27.294)	<0.001
Dyspnea	38 (23.9)	23 (74.2)	15 (11.7)	20.612 (7.722–55.022)	<0.001
Tract bleeding	23 (14.5)	10 (32.3)	13 (10.2)	3.874 (1.427–10.514)	0.008
Drowsiness	36 (22.6)	14 (45.2)	22 (17.2)	4.180 (1.691–10.332)	0.002
Neural trance	29 (18.2)	15 (48.4)	14 (10.9)	6.701 (2.679–16.762)	<0.001
Conscious disturbance	25 (15.7)	15 (48.4)	10 (7.8)	11.879 (4.315–32.703)	<0.001
**Viral load(log10copies/mL)**					
Mean ± SD	4.07±1.44	5.29±1.49	3.80±1.30	2.544 (1.429–4.528)	0.002
**Laboratory index (Mean ± SD)**					
*WBC (×10^9^/L)	4.23±3.82	5.34±6.04	3.98±3.11	1.077 (0.986–1.177)	0.100
*PLT (×10^9^/L)	48.29±26.26	37.66±26.94	50.61±25.61	0.978 (0.959–0.998)	0.034
RDW (%)	13.61±0.97	13.96±0.89	13.54±0.97	2.440 (1.392–4.278)	0.002
*APTT (secs)	43.76±14.27	54.30±18.10	41.60±12.35	1.057 (1.025–1.090)	<0.001
*DD (ng/ml)	1571.38±1990.26	2372.79±3064.05	1392.05±1620.83	1.000 (1.000–1.000)	0.016
*CKMB (U/L)	47.84±60.26	102.35±102.85	37.36±40.87	1.012 (1.005–1.020)	0.001
*LDH (U/L)	915.99±725.94	1695.11±1055.50	783.99±559.57	1.002 (1.001–1.003)	<0.001
*AST (U/L)	372.62±501.13	645.29±570.40	313.34±465.97	1.001 (1.000–1.003)	0.002
*AST/ALT	3.05±1.95	4.66±2.92	2.72±1.48	1.597 (1.249–2.042)	<0.001
*DBIL (μmol/L)	8.58±14.28	15.55±29.05	7.05±7.47	1.035 (1.002–1.069)	0.040
ALB (g/L)	29.64±3.83	27.87±3.70	30.04±3.76	0.8242 (0.749–0.946)	0.004
GGT (U/L)	106.10±135.19	174.81±153.98	91.54±126.71	1.004 (1.001–1.007)	0.004
*ALP (U/L)	105.24±80.87	149.50±102.97	95.85±72.37	1.006 (1.002–1.011)	0.005
*TBA (μmol/L)	13.42±29.62	32.97±61.88	9.04±11.61	1.031 (1.005–1.058)	0.021
*CREA (μmol/L)	95.66±87.79	148.06±139.84	84.20±66.98	1.007 (1.002–1.012)	0.010
*UA (μmol/L)	288.77±157.41	408.44±237.97	262.84±119.89	1.006 (1.003–1.009)	<0.001
*CO2 (mmol/L)	21.01±4.93	17.82±5.75	21.74±4.44	0.838 (0.761–0.923)	<0.001
*IL-6 (pg/mL)	220.06±664.85	841.84±1358.41	77.93±162.25	1.003 (1.001–1.005)	0.004
*CRP (mg/L)	20.71±41.40	48.61±92.40	16.44±24.65	1.014 (1.000–1.027)	0.048
SAA (mg/L)	127.45±101.18	223.26±96.00	108.75±91.57	1.011 (1.004–1.019)	0.002
HDL (mmol/L)	0.67±0.32	0.40±0.22	0.72±0.32	0.001 (0.000–0.051)	<0.001

Statistical analyses of categorical variables including sex, underlying diseases and clinical manifestations were performed using χ^2^ test and Fisher’s exact test. Statistical analyses of continuous variables including age, days since symptom onset, viral load and laboratory index were performed using one-way ANOVA.

“*” in laboratory index used non-parameter Kruskal-Wallis test. p <0.05 was considered statistically significant.

### Genetic diversity shifts of SFTSV circulating in Hubei Province

**Fig 2:**
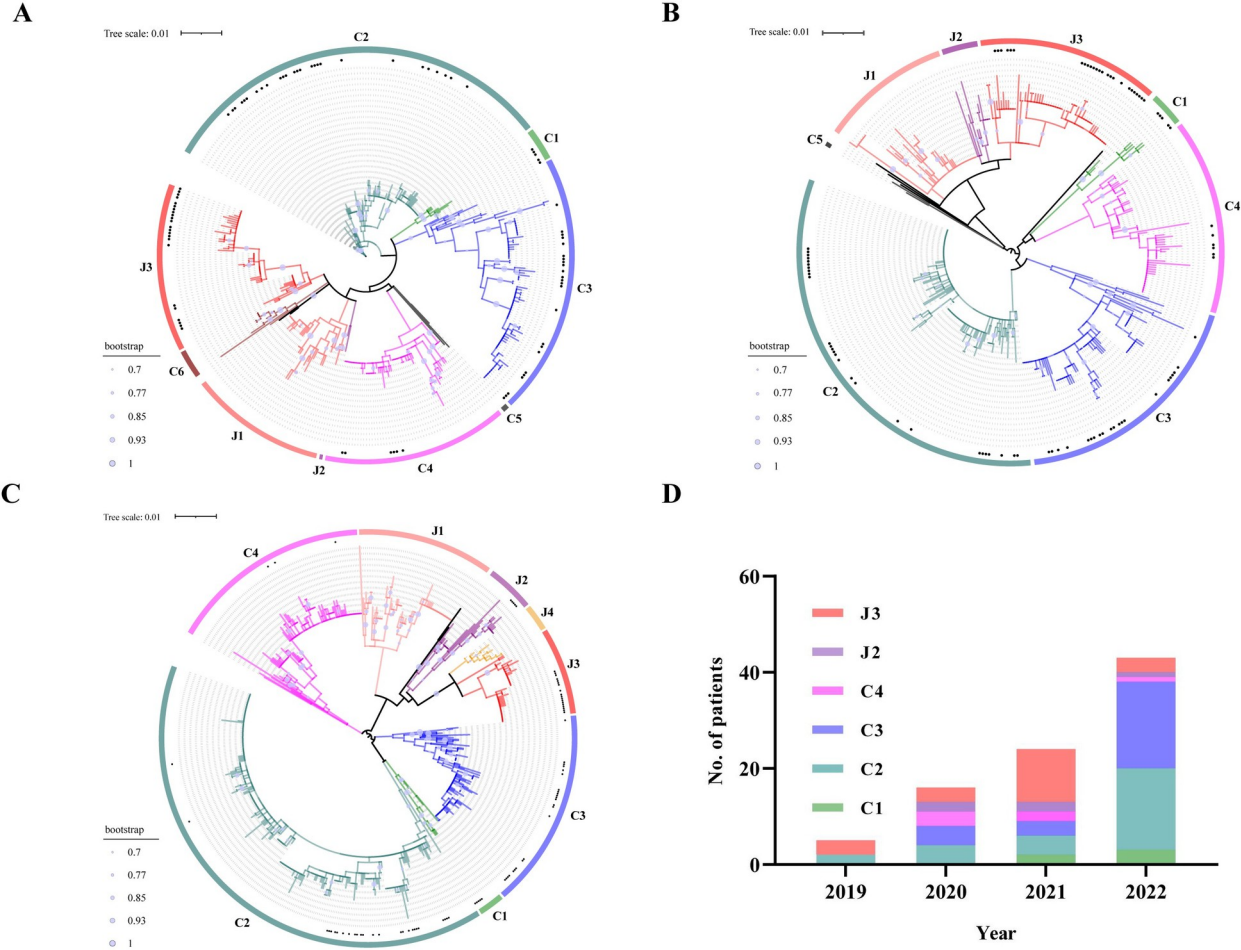
Phylogenetic relationships of the partial sequence of L, M and S segments of SFTSV strains. The ML trees were constructed based on the partial sequence alignment of L (700bp, A), M (500bp, B), and S (770bp, C) segments. The SFTSV strains detected in this study are indicated by bold black spots. The ML trees were constructed and tested by bootstrap analysis with 1000 iterations and only bootstrap values above 70% are shown. Ten genotypes, colored in pink, purple, red, yellow, green, cyan, blue, fuchsia, gray and brown evolutionary branches were designated as genotype J1, J2, J3, J4, C1, C2, C3, C4, C5 and C6, respectively. (D) The number and proportion of different genotypes obtained from patients with SFTS in each studied year.

Since many patients were transferred to Zhongnan Hospital from local hospitals, serum collection of some patients was conducted in the late disease period when the viral load may have been below the detection limit. Therefore, we finally obtained L, M, and S sequences of SFTSV from 88 SFTS patients’ serum using the nested RT-PCR in laboratory. Phylogenetic analysis was performed based on the L, M, and S partial sequences of the 88 SFTSV strains detected in this study and all available SFTSV sequences in GenBank. It revealed that the total SFTSVs can be divided into eight normal genotypes (C1-C5 in Chinese lineage and J1-J3 in Japanese lineage) with two potential novel sub-genotypes (C6 and J4) based on classification methods described in previous study [9] (Fig 2). Our study indicated that a total of 6 SFTSV genotypes including C1-C4 and J2-J3, were circulated in Hubei Province.

When using S segment as typing gene, the most prevalent genotype circulated in Hubei Province was C2 (32.1%), followed by genotype C3 (28.4%) and genotype J3 (22.2%). While the proportion of genotype C1, J2 and C3 was 6.2%, 6.2% and 4.9%, respectively. Significantly, the SFTSV strains firstly identified in Hubei Province in 2011 all belonged to genotype C3 [10], but in recent four years (2019–2022), its prevalence was lower than genotype C2. Meanwhile, the dominant genotype of SFTSV was changing during the years, as genotype J3 was the most prevalent in 2019–2021, but the dominant genotype changed to genotype C3 and genotype C2 in 2022 (Fig 2D). These results revealed the complex genotype diversity of SFTSV prevalent in Hubei Province.

In addition, the SFTSV detected in this study were mainly distributed in the Dabie Mountains region with a high prevalence rate and complex genotype diversity (S2 Fig), suggesting that it was necessary to reinforce the surveillance of SFTS outbreaks in these areas and investigate the variation and evolution of virus strains, which will be beneficial to the prevention and control of SFTS in Hubei Province.

### Phylogenetic analysis of SFTSV strains indicated potential reassortment events

**Fig 3:**
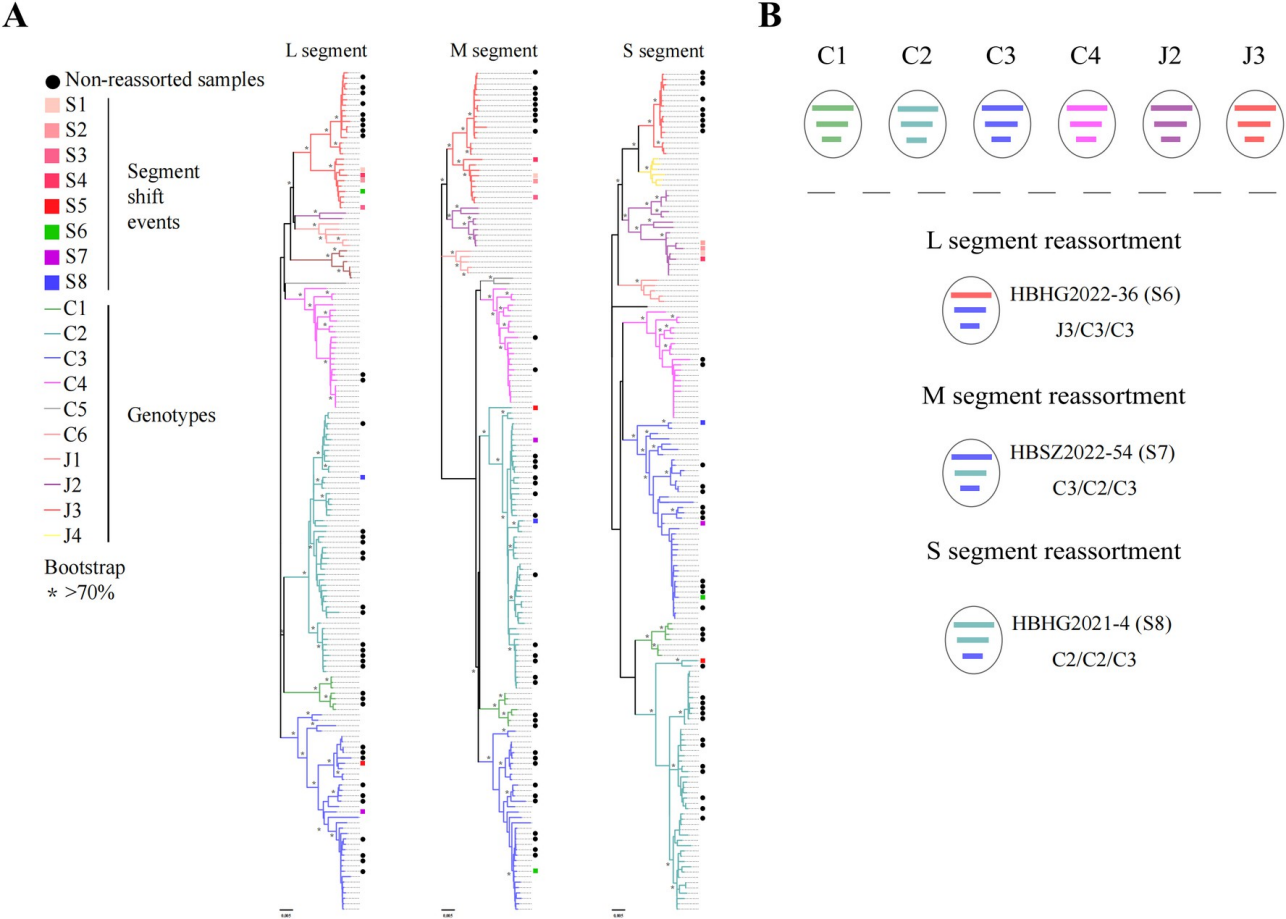
Phylogenetic analysis based on the complete ORF sequences of L, M and S gene segments of Hubei SFTSVs. (A) Segment shift events were specially marked including S1 (HBHG2020-02), S2 (HBHG2020-08), S3 (HBHG2021-02), S4 (HBHG2022-12), S5 (HBSZ2022-11), S6 (HBHG2022-36), S7 (HBSZ2022-54) and S8 (HBHG2021-4). The bars indicate pure groups of genotypes colored with green (C1), cyan (C2), blue (C3), fuchsia (C4), gray (C5), brown (C6), pink (J1), purple (J2), red (J3), yellow (J4). The ML trees were constructed and tested by bootstrap analysis with 1000 replications, and these phylogenic trees were mid-point rooted. The scale bars (0.005) indicate the number of nucleotide substitutions per site. “*” representing that the phylogenetic branches were supported with greater than 70% bootstrap values. (B) Genotypes of Hubei SFTSVs detected in human sera and the graphic representation of reassortment events in SFTSV genome sequences obtained from patients. The SFTSVs were assigned to different genotypes based on the genetic origin of each segment as determined by phylogenic analysis. Gene segments from top to bottom are L, M, and S.

To further investigate the characteristic of genetic variation and deep evolutionary relationships, 45 SFTSV full-length sequences from SFTS patients were obtained in this study. The L, M, and S trees presented similar topological structures according to the phylogenetic analysis of whole genome segments of 45 SFTSVs in Hubei Province (Fig 3A, detailed in S3 Fig). The majority of SFTSVs were clustered into the same genotype in each of the L, M and S trees based on complete sequences. Intriguingly, phylogenetic analysis also exhibited that eight SFSTV stains identified in our study showed potential reassortment events as they shifted the evolutionary positions among L, M and S phylogenetic trees, involving five in S segment, one in M segment, and two in L segment (Fig 3B). For example, the M and S segments of strain HBHG2022-36 fell into the C3 genotype, while its L segment clustered with the J3 genotype. HBHG2020-02, HBHG2020-08, HBHG2021-02 and HBHG2022-12 were the dominant potential reassortment strains, where both L and M segments of these four strains were grouped with J3 genotype, whereas S segment belonged to J2 genotype. These results suggested that the Hubei SFTSV strains may have a multiformity of evolutional reassortment pattern.

### Reassortment and recombination events

A total of three SFTSV reassortment strains were finally confirmed using RDP4 software to analyze the concatenated sequence of L, M and S, including HBHG2021-4 (C2/C2/C3), HBHG2022-36 (J3/C3/C3) and HBSZ2022-54 (C3/C2/C3). Obviously, genotype C3 existed in different gene segments of the SFTSV recombinant strains founded in this study, indicating that genotype C3 have the highest possibility to participate in reassortment event. Further, we found the occurrence of reassortment event in different areas in Hubei Province, or with adjacent province (Henan) by analyzing the parental strain of these (S5 Table). The result revealed that genetic exchange of SFTSV in Hubei Province or between other province enriched its genetic diversity.

In particular, recombination signals were also detected in Hubei SFTSV strains using RDP4 software package. Based on different regions of L, M and S segment delimited by potential breakpoint(s) listed in S6 Table, a total of eight SFTSV strains were proved to possess recombination events using Simplot software and inconsistent phylogenetic analysis. Half of the eight recombinational SFTSV strains involved M segment, while three to the S segment and only one to the L segment. It is also noteworthy that multiple recombination events existed in HBSZ2022-11 with potential reassortment event, and it was distributed in independent branch apart from other genotype C3 strains in M phylogenetic tree. These results demonstrated that recombination may affect the changing of SFTSV genotype, thus leading to the production of new genotypes in SFTSV. In summary, the genetic exchange of Hubei SFTSV strains was relatively frequent, which may affect the continuous variation of SFTSV epidemic characteristics in Hubei Province.

### Correlation between the disease severity and SFTSV genetic variants

**Fig 4:**
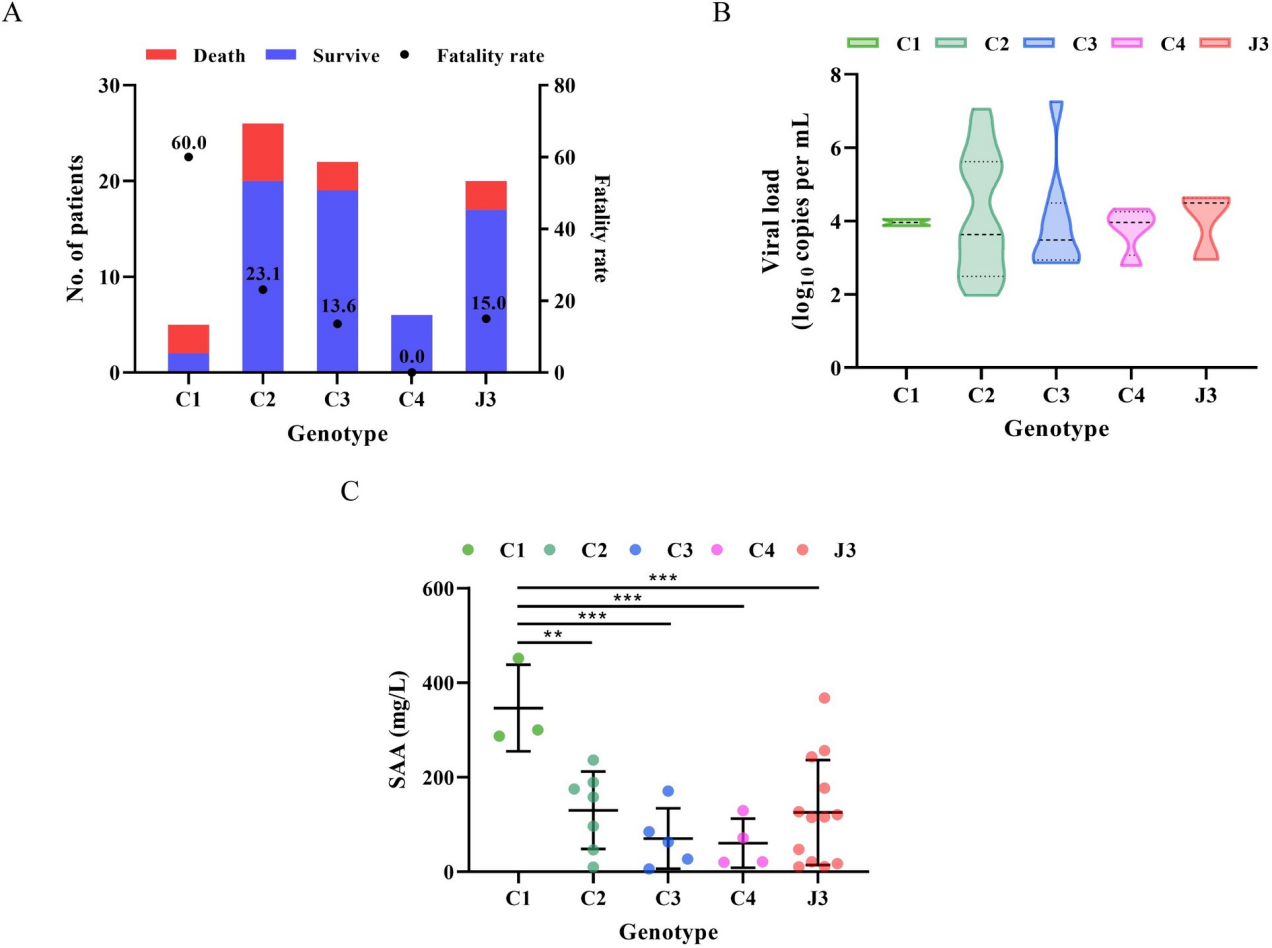
Fatality rates, viral loads and laboratory index of patients infected with five viral genotypes. (A) Red columns represent dead patients and blue column represent survival patient. Datapoints show CFRs; error bars show 95% CIs; (B) Horizontal and dotted lines indicate the mean value and IQR. The statistical significance (p<0.05) of the comparison of viral load among these five genotypes used the ANOVA test. (C) Intermediate lines show mean value; error bars show standard deviation (SD). The star indicates the statistical significance (*p*<0.05) of the comparison of laboratory variables between the patients infected with genotype C1 and other four genotypes determined by the ANOVA test. “*” indicate 0.01<*p*<0.05, “**” indicate 0.001<*p*<0.01, “***” indicate *p*<0.001 (detailed in S8 and S9 Tables).

To further investigate the influence of genetic variation characteristics on the pathogenicity of SFTSV, we compared case fatality rate (CFR) and viral load in *vivo* among patients infected with different genotypes of SFTSV. The CFR of genotype C1 reached the highest level (60%), compared to other genotypes (Fig 4). However, there was no statistical difference in CFR and viral load among different genotypes, possibly due to the limited number of identified SFTSV sequences. Further, we found genotype C1 associated with a higher risk of death (vs. genotype C2) after adjusting age, sex and comorbidities based on multivariate logistic regression analysis (S7 Table).

Besides, we assessed clinical manifestations and laboratory variables that were considered to be correlated to fatal consequence of SFTS patients infected with different viral genotypes (S8 Table). It was obviously observed that conscious disturbance (*p* = 0.005), direct bilirubin (*p* = 0.002), alkaline phosphatase (*p* = 0.014), γ-glutamyl transpeptidase (*p* = 0.037) and serum amyloid A (*p* = 0.003) showed significant difference among these five viral genotypes (C1, C2, C3, C4 and J3). Moreover, the level of serum amyloid A (SAA), a laboratory variable related to mortality, was the highest in SFTS patients infected with genotype C1 (Fig 4C and S8 Table). It was reported that SAA mediated the dysfunction and activation of endothelial, stimulated platelet aggregation and degranulation leading to hemostatic failure, resulting in consequent activation of the intrinsic coagulation cascade, which was consistent with the late progress of disseminated intravascular coagulation (DIC) in most of the severe and fatal SFTS cases [25]. These findings collectively suggested that one specific viral genotype (C1) may have a tendency to cause virus-induced hyperinflammation and result in higher fatality rates.

## Discussion

The continuous infection of SFTSV to human and high case fatality rate of SFTS have caused a wide range of public concern. In this study, we investigated the clinical characteristic of SFTS patients and genetic features of SFTSV with long time span (2018–2022, 5 years) in Hubei Province. The locational origin of SFTS patients characterized as broad coverage, with most of epidemic regions in Hubei Province were involved in our study, although patients were recruited at a single center. This research can representatively reflect the clinical characteristic of SFTS and the changing epidemiological features along with genetic variation characteristics of SFTSV in epidemic region. On top of that, we preliminarily analyzed the correlation between the SFTS disease severity and SFTSV genetic variants.

Firstly, we found a higher risk of death in Hubei SFTS patients who present multiorgan failure, dyspnea, tract bleeding, drowsiness, neural trance and conscious disturbance compared to those without these symptoms. This is consistent with the findings of Li et al. [18], who found that patients with more neurological symptoms had a higher risk of fatal outcomes. Meanwhile, a total of 20 laboratory indicators significantly correlated with fatal consequence across hospitalization in our study. Zhao J et al. [26] also demonstrated that 11 laboratory indexes continuously deviated from normal range during hospitalization. However, the limitation of our study is that we lacked the dynamic analysis of clinical data, which limits further analysis of critical points in clinical deterioration. Taken together, it is necessary to enhance the surveillance, vigilance, and awareness of the abnormal fluctuation of these important manifestations, index and viral load to prevent rapid deterioration in time.

SFTSV strains were originally clustered into two clades (Chinese lineage and Japanese lineage) including 8 genotypes, indicating that SFTSV evolution was associated with geographic distribution based on segments analysis [11]. Reassortment and recombination events were discovered in numerous SFTSV strains. Initially, Hubei SFTSV strains were found to clustered in only one genotype (C3) and were considered to be separated from other regions [10]. However, subsequent studies showed the simultaneous prevalence of C2 and C3 genotypes in SFTSVs in Hubei Province [17]. In this study, we found the co-circulation of 6 different genotypes of SFTSV Hubei strains based on S segment, with variational dominant genotypes in different years, indicating that the genetic diversity of the epidemic area has increased.

Like other segmented genomic virus, reassortment event have been reported to occur in SFTSV isolated from patients, ticks and animals [11,12,27]. In a recent study, 14 SFTSV strains were observed to exhibit potential reassortment events, with 13 events involving the L segment and 1 event involving the M segment [16]. In our study, a total of 8 potential reassortants were found, with 3 of them were further identified using RDP4 software. The reassortment strains of SFTSV from the same region (Hubei) were firstly found to involve all three segments with 1 derived from reassortment of segment L, 1 of segment M, and 1 of segment S. This result indicated increased genetic exchange among Hubei SFTSV strains, which could be the result of the epidemic and spread of the virus across regions. Based on migration analysis of SFTSV in 2021, Hubei Province was found to be the key area for the migration of multiple genotypes of virus and genotype C2, C3 and C4 had more migration paths [12]. While in this study, reassortants mostly belonged to genotype C2, C3 and J3, indicating diversity and variability of migration paths. Additionally, it is speculated that the cross-regional dissemination migration of SFTSV strain may be related to the migration of birds in recent studies.

Inter-gene recombination plays an important role in the rapid genetic evolution of segmental virus, and natural recombination events were also found in L, M and S fragments of SFTSV [28]. In this study, we identified a total of 8 novel recombination events in Hubei SFTSV strains from patients referring to all segments with half of which focus on M segment. It was reported that the virus immunogenicity and neutralizing or protective epitopes was contained by glycoprotein encoded by the M segment of bunyaviruses [29]. The involvement of M segment in most of recombination events we observed, suggesting that antigenic shift may be related with recombination within M segments. Additionally, we found that multiple recombination events occurred in a Hubei SFTSV strain (HBSZ2022-11), which indicated an evolutionary position change in phylogenetic tree suggesting that it was a potential reassortant, distributed in independent branches as well. These results unveiled that Hubei Province played a pivotal role in the gene reassortment or recombination of SFTSV strain and demonstrated that recombination may influence the changing of SFTSV genotype, thus leading to the production of new genotypes in SFTSV, and further cause the change of antigenic sites or antigenic drift of the viral strains.

Comparison of genetic sequence identity suggests that strains belonging to the same genotype have similar genetic variation characteristics. Wu et al. [12] found that genotypic SFTSV strains also had genotypic specific amino acid mutations, suggesting that the evolution of different genotypes may affect the infectivity, replication efficiency and pathogenicity of the virus, which should be further studied and analyzed. CFR could be considered as an indicator of the SFTSV virulence in patients treated with the same standard regimen. Yun et al. [15] conducted an analysis of the full-length sequence of SFTSV isolated from serum of 116 SFTS patients in South Korea in 2019. They found that SFTSV strains in genotype B (equivalent to Japanese lineage) had the highest case fatality rate compared to other genotypes and the virus had different pathogenicity potential with specific genotypic according to ferret infection research. In 2022, Dai et al. [16] assessed the correlation between the characteristics of genetic variation and clinical data in more than 800 SFTSV strains in Xinyang, Henan Province. They revealed that clade IV (belong to genotype C3) exhibited a significantly higher CFR with 32.9% and found a significant correlation between one specific viral clade and SFTS fatality through molecular and immunological studies. In our study, the Hubei SFTSV strains with the highest CFR (60%) belonged to genotype C1(corresponding to clade V). These results suggested that the influence of viral genotypes to disease fatality and severity may be distinct due to regional distribution and individual differences of patients. However, only a limited number of SFTSV sequence were identified within each viral genotype, resulting in no significant differences of the CFR and viral load among different viral genotypes. Thus, it is difficult to ensure whether there is a correlation between different genotypes and mortality in Hubei SFTSV strains.

Further, we found that SFTS patients infected with genotype C1 exhibited significantly higher level of SAA than other four viral genotypes, suggesting that genotype C1 may induce a stronger virus-induced hyperinflammatory response in patients. Therefore, it is suggested to conduct further research in the effect of the genetic variation characteristics to the lethality of SFTSV by recruiting a large case-cohort of SFTS patients with the same treatment in endemic regions of SFTS. Besides, IL-6 exhibited significantly higher level in fatal (severe) cases than survival (mild) cases in our study and previous studies, indicating that the imbalance of cytokine and chemokine production can lead to cytokine-mediated inflammatory response and might further cause a fatal case of SFTS [30]. Whereas, there was no significant difference in IL-6 level among different genotypes of SFTSV in this study, which is related to the involvement of multiple factors in different clinical outcomes of SFTS. To identify the virulence-affecting mutations, reverse genetics analysis should be combined with experiments on the cellular and animal level. Choi et al. [31] discovered the mutation at the amino acid position 102 from proline to alanine in NS protein (P102A) could inhibit the production of IL-10, thus improving the survival rate of IFNAR−/− mice infected with SFTSV. Apart from genetic variational characteristic, the underlying disease, age, clinical manifestation and individual immunity level of the host resulting in cytokine storm might be correlated with the severity of SFTS as well. Further research should focus on whether the virus-host interaction and the factors of the host itself lead to different disease outcomes based on single-cell RNA sequencing (scRNA-seq) and other approaches, thus laying the foundation for the prevention and control of SFTSV strain and the research on vaccine and drugs.

Overall, the results from this study surveyed the clinical characteristic of SFTS patients in Hubei Province from 2018 to 2022 and tracked the epidemic and genetic variational features of SFTSV strains detected from these patients’ serum. Thus, we further analyzed the epidemic evolutionary characteristics and trends of SFTSV strains in recent years, providing an important experimental basis for the development of SFTS prevention, control and treatment strategies in Hubei Province. Meanwhile, we clarified the status of SFTSV genetic variation in Hubei Province and preliminarily analyzed the CFR, viral load and clinical data of different viral genotypes, which enabled us to identify high virulent SFTSV strains in the region. This study also provides a foundation for future studies on the correlation between SFTSV genetic variation characteristics and disease severity.

## Supporting information

S1 Table Primers used for nested RT-PCR assays. (PDF)

S2 Table SFTSV sequences determined in the current study with the name of isolate and corresponding accession number. (PDF)

S3 Table SFTSV sequences of L, M and S available from GenBank were listed with name of isolate, location and accession number. (PDF)

S4 Table Association of all demographic, clinical variables and clinical manifestations with fatal case in the patients evaluated with the use of logistic regression model by adjusting sex and delay from onset to admission. (PDF)

S5 Table Confirmation of the SFTSV segment reassortment by seven methods using RDP packages. (PDF)

S6 Table SFTSV recombination events detected using the RDP package. (PDF)

S7 Table Logistic Regression Analysis of Variables Associated with Mortality. (PDF)

S8 Table Laboratory index and presence of severe clinical manifestations during hospitalization among SFTS patients infected with different viral genotypes. (PDF)

S9 Table Comparison of mortality-associated laboratory variables between patients infected with Genotype C1 and each of other four viral genotypes by One-Way ANOVA test. (PDF)

S10 Table Acronym list. (PDF)

S1 Fig CFR and adjusted OR for death of patients with SFTS by clinical manifestations. The black points are the adjusted ORs for death and the black error bars are the 95% CIs. ORs were adjusted for age, sex, and delay from symptom onset to admission. The adjusted OR for macroscopic hematuria have a higher scale than the other symptoms. The dotted line indicates an adjusted OR of 1. CFR = case fatality rate. OR = odds ratio.
(PDF)

S2 Fig Location information and genotype of SFTS patients. The map was created in ArcGIS 10.2 software (ESRI Inc., Redlands, CA, USA) and modified using Adobe illustrator, Version CC2018 (Adobe, San Jose, CA, USA). The source of the base layer shapefile was from the open access platform: National Platform for Common Geospatial Information Services (www.tianditu.gov.cn).
(PDF)

S3 Fig The full version of the Maximum Likelihood (ML) trees of Fig 3. Phylogenetic analysis based on the complete ORF sequences of (A) L, (B) M, and (C) S segments of Hubei SFTSVs and all available reference sequences. The full version of the Maximum Likelihood (ML) trees based on the Kimura 2-parameter model were constructed and tested by bootstrap analysis with 1,000 replications. The scale bar indicates the number of nucleotide substitutions per site, and the phylogenetic branches were supported with greater than 70% bootstrap values.
(PDF)
