# Supplementary material for: Extensive genetic diversity of severe fever with thrombocytopenia syndrome virus circulating in Hubei Province, China, 2018–2022
Source: PLoS Negl Trop Dis. 2023 Sep 18;17(9):e0011654. doi: 10.1371/journal.pntd.0011654 (PMC10538666; doi:10.1371/journal.pntd.0011654)
Supplement: S1 Table — (PDF) [file pntd.0011654.s001.pdf]

S1 Table. Primers used for nested RT-PCR assays.

Segment	First round		Second round	
	Primer name	Primer sequence (5'-3')	Primer name	Primer sequence (5'-3')
L	L1F	ACACARARAMGCCCAGATGRACTTG	LN-1F	CACARARAMGCCCAGATGRACTTGG
	L1R	CAGGATGAGGCWGAGGAGCT	LN-1R	GRGACCCAATCTCAAGRCGTGTTG
	L2F	ATAGGGGYTGTGCCRGACTCDG	LN-2F	GACWACAGAYCGACGYCTMTCAGAG
	L2R	CCGCAAGGCYGAYGTGACAAG	LN-2R	TCATCAAGAAGCTGAARTTCTGGCC
	L3F	AGYGACCCWGCAAAAGARCTTGRC	LN-3F	ACCCCYARRGAGGCHAAGATAACC
	L3R	AGCTCTACTCAGAAGTCCARACMAA	LN-3R	GACCTTGCATCBATGAARGCYACAA
	L4F	TGCAGTTTGGSGTYGAGACCATG	LN-4F	AGGTGTGTYTGTGAGCTRAGCC
	L4R	CATGAARCAYGGGGGCTCACT	LN-4R	GTGGTGGCAGCAGGRGTRTA
	L5F	GATATCAGACCCAGATCCWGCCC	LN-5F	TCCTCATGGACARCCCTGCA
	L5R	GAGGAGATCAGGCTCAGTAGTGAT	LN-5R	GGACTGGRGTGATGGARGAYACC
	L6F	GGAACTTCATAGCCCAYGTTGATGC	LN-6F	GAARTCAGGHGGGGTCACCAC
	L6R	GARGAGAGCACYATYATGTGGGGTT	LN-6R	CAATGTGTGCRATCATGGGYAAGG
	L7F	CTCTGGAGYAATMGAGACCTCTTCT	LN-7F	GGCAAATGATAGGAAGACCCAAGG
	L7R	AGATCTGGGCGGTCTTTGTGT	LN-7R	TGTGTCTGTGGGTGRCYARGGA
M	M1F	CACARTAGRCGGCCAACAATGATG	MN-1F	AGRCGGCCAACAATGATGAAAGTC
	M1R	ATGAGGGAGCACAAGACCAAATG	MN-1R	GCRTGGATGGATGTWGGYCACTC
	M2F	TGTGGYAGTGGGAAGTCWAGRA	MN-2F	ATAGGGTTCTCTGGATAGGTGATGT
	M2R	GATGGATAGRGGAAGGCAAGTGATC	MN-2R	TGGGTVATRGCTCCAGTGAAGC
	M3F	CRGTGGAYTGCACWTTCTGTCG	MN-3F	CAGTGCTACCCTGCAAAGAARTG
	M3R	GGCAAGGTTGGAGAGATACAGTG	MN-3R	ACARGTYTTCAAGGGTGTGAGTGT
	M4F	GCRGTGAACCCAGGDCAAGAG	MN-4F	CTGCACTTCACRGCRCCTGG
	M4R	GCAGWGCTCTCAGGGGAGT	MN-4R	GGCAATGGGCTRAGBAGRTGG
	M5F	GCATGACAGGYGCCAAAGTCTC	MN-5F	TTCAGTGTCYTGGARGGRGTYCA
	M5R	GTGTTGGCCGGTCTTTGTGT	MN-5R	GRAGTGTTGGCCGGTCTTTGT
S	S1F	ACACAAAGAAMCCCCTTCATTTGGA	SN-1F	CAAAGAAMCCCCTTCATTTGGAAAC
	S1R	GTAAGCAGCAGCAGCAACCT	SN-1R	CRGCYATTGGTGACCYCCCRAAG
	S2F	GGCCCTCCTYCAGATAGAGTCAC	SN-2F	CTTCTYCCTCRTTGCGYAAGCCTCT
	S2R	GCTGCTGCTCWCCAAACTYCAC	SN-2R	GGCCYTCATARGCYAGCTCTCTCG
	S3F	GGCCATGCACATCATCTCWGG	SN-3F	GAGYCTRGTYTCTGCCCTCTCAAC
	S3R	GCGTCTTTCCTTTTTTGGGGG	SN-3R	CATGWTCWCTCCTWTRCGTCTTTCC
