# Supplementary material for: Extensive genetic diversity of severe fever with thrombocytopenia syndrome virus circulating in Hubei Province, China, 2018–2022
Source: PLoS Negl Trop Dis. 2023 Sep 18;17(9):e0011654. doi: 10.1371/journal.pntd.0011654 (PMC10538666; doi:10.1371/journal.pntd.0011654)
Supplement: S2 Table — (PDF) [file pntd.0011654.s002.pdf]

S2 Table. SFTSV sequences determined in the current study with the name of isolate and corresponding accession number.

Segment/Accession No.	L		M		S	
	Partial sequence	Complete sequence	Partial sequence	Complete sequence	Partial sequence	Complete sequence
HBHG2019-01/Huanggang_Yingshan/2019			OQ389001		OQ388871	
HBHG2019-04/Huanggang_Hongan/2019	OQ388786					
HBHG2019-09/Huanggang_Qichun/2019	OQ388787	OQ388741	OQ389002	OQ389082	OQ388872	OQ388955
HBHG2019-10/Huanggang_Yingshan/2019	OQ388788	OQ388742	OQ389003	OQ389083	OQ388873	OQ388956
HBHG2019-12/Huanggang_Yingshan/2019	OQ388789		OQ389004		OQ388874	
HBHG2020-01/Huanggang_Xishui/2020	OQ388790	OQ388784	OQ389005	OQ389084	OQ388875	OQ388999
HBHG2020-02/Huanggang_Qichun/2020	OQ388791	OQ388785	OQ389006	OQ389085	OQ388876	OQ388998
HBHG2020-03/Huanggang_Yingshan/2020	OQ388792	OQ388743	OQ389007	OQ389086	OQ388877	OQ388957
HBHG2020-04/Huanggang_Macheng/2020	OQ388793		OQ389008		OQ388878	
HBHG2020-05/Huanggang_Hongan/2020	OQ388794		OQ389009		OQ388879	
HBHG2020-08/Huanggang_Yingshan/2020	OQ388795	OQ388744	OQ389010	OQ389087	OQ388880	OQ388958
HBHG2020-11/Huanggang_Yingshan/2020	OQ388796	OQ388783	OQ389011	OQ389088	OQ388881	OQ388997
HBHG2020-12/Huanggang_Hongan/2020	OQ388797	OQ388745	OQ389012	OQ389089	OQ388882	OQ388959
HBHG2020-14/Huanggang_Yingshan/2020	OQ388798		OQ389013	OQ389090	OQ388883	OQ388996
HBXG2020-16/Xiaogan_Dawu/2020	OQ388799	OQ388781	OQ389071	OQ389118	OQ388884	OQ388995
HBHG2020-17/Huanggang_Hongan/2020	OQ388800		OQ389014		OQ388885	
HBHG2020-20/Huanggang_Hongan/2020	OQ388801		OQ389015		OQ388886	
HBHG2020-21/Huanggang_Qichun/2020	OQ388802		OQ389016		OQ388887	
HBHG2020-22/Huanggang_Luotian/2020	OQ388803		OQ389017		OQ388888	
HBHG2020-24/Huanggang_Hongan/2020	OQ388804				OQ388889	
HBXG2020-26/Xiaogan_Dawu/2020	OQ388805	OQ388746	OQ389072	OQ389119	OQ388890	OQ388960
HBHG2021-01/Huanggang_Qichun/2021	OQ388806	OQ388780	OQ389018	OQ389091	OQ388891	OQ388993
HBHG2021-02/Huanggang_Luotian/2021	OQ388807	OQ388747	OQ389019	OQ389092	OQ388892	OQ388961
HBHG2021-03/Huanggang_Qichun/2021	OQ388808		OQ389020		OQ388893	
HBHG2021-04/Huanggang_Wuxue/2021	OQ388809	OQ388748	OQ389021	OQ389093	OQ388894	OQ388962
HBXG2021-05/Xiaogan_Dawu/2021	OQ388810					
HBXG2021-08/Xiaogan_Dawu/2021	OQ388811				OQ388895	
HBWH2021-09/Wuhan_Xinzhou/2021	OQ388812	OQ388779	OQ389067	OQ389117	OQ388896	OQ388992
HBHG2021-11/Huanggang_Yingshan/2021	OQ388813				OQ388897	
HBXG2021-12/Xiaogan_Dawu/2021	OQ388814				OQ388898	
HBHG2021-13/Huanggang_Yingshan/2021	OQ388815	OQ388749	OQ389023	OQ389098	OQ388899	OQ388963
HBWH2021-16/Wuhan_Xinzhou/2021	OQ388816		OQ389068		OQ388900	
HBHG2021-18/Huanggang_Yingshan/2021	OQ388817	OQ388778	OQ389024	OQ389095	OQ388901	OQ388994
HBHG2021-19/Huanggang_Qichun/2021					OQ388902	
HBHG2021-W2/Huanggang_Yingshan/2021	OQ388818		OQ389029		OQ388903	
HBHG2021-W3/Huanggang_Yingshan/2021	OQ388819	OQ388760	OQ389030	OQ389094	OQ388904	OQ388980
HBHG2021-W7/Huanggang_Yingshan/2021	OQ388820		OQ389031		OQ388905	
HBHG2021-W8/Huanggang_Yingshan/2021			OQ389032		OQ388906	
HBHG2021-W11/Huanggang_Yingshan/2021	OQ388821	OQ388750	OQ389027	OQ389097	OQ388907	OQ388964
HBHG2021-W17/Huanggang_Yingshan/2021	OQ388822		OQ389028		OQ388908	
HBXG2022-01/Xiaogan_Dawu/2022	OQ388823	OQ388776	OQ389073	OQ389120	OQ388909	OQ388991
HBHG2022-02/Huanggang_Luotian/2022	OQ388824		OQ389033		OQ388910	
HBXG2022-03/Xiaogan_Dawu/2022			OQ389074			
HBXG2022-04/Xiaogan_Dawu/2022	OQ388825	OQ388775	OQ389075	OQ389121	OQ388911	OQ388990
HBHG2022-05/Huanggang_Qichun/2022	OQ388826		OQ389034			
HBXG2022-06/Xiaogan_Dawu/2022	OQ388827		OQ389076		OQ388912	
HBXG2022-07/Xiaogan_Xiaochang/2022	OQ388828	OQ388751	OQ389077	OQ389122	OQ388913	OQ388967
HBXG2022-08/Xiaogan_Xiaochang/2022	OQ388829	OQ388774	OQ389078	OQ389123	OQ388914	OQ388989

HBSZ2022-17/Suizhou/2022	OQ388830		OQ389047		OQ388915	
HBSZ2022-18/Suizhou/2022	OQ388831	OQ388773	OQ389048	OQ389107	OQ388916	OQ388988
HBSZ2022-19/Suizhou/2022	OQ388832		OQ389049		OQ388917	
HBSZ2022-20/Suizhou/2022	OQ388833		OQ389050		OQ388918	
HBSZ2022-21/Suizhou/2022	OQ388834		OQ389051		OQ388919	
HBSZ2022-22/Suizhou/2022	OQ388835	OQ388772	OQ389052	OQ389108	OQ388920	OQ388987
HBSZ2022-23/Suizhou/2022	OQ388836	OQ388771	OQ389053	OQ389109	OQ388921	OQ388986
HBSZ2022-24/Suizhou/2022	OQ388837		OQ389054		OQ388922	
HBSZ2022-25/Suizhou/2022	OQ388838	OQ388770	OQ389055	OQ389110	OQ388923	OQ388985
HBSZ2022-26/Suizhou/2022	OQ388839		OQ389056		OQ388924	
HBSZ2022-27/Suizhou/2022	OQ388840	OQ388752	OQ389057	OQ389111	OQ388925	OQ388965
HBSZ2022-28/Suizhou/2022	OQ388841	OQ388769	OQ389058	OQ389112	OQ388926	OQ388984
HBSZ2022-29/Suizhou/2022	OQ388842	OQ388768	OQ389059	OQ389113	OQ388927	OQ388983
HBSZ2022-30/Suizhou/2022	OQ388843	OQ388767	OQ389060	OQ389114	OQ388928	OQ388975
HBSZ2022-31/Suizhou/2022	OQ388844	OQ388766	OQ389061	OQ389115	OQ388929	OQ388982
HBSZ2022-32/Suizhou/2022	OQ388845		OQ389062		OQ388930	
HBSZ2022-33/Suizhou/2022	OQ388846		OQ389063		OQ388931	
HBSZ2022-34/Suizhou/2022	OQ388847		OQ389064		OQ388932	
HBSZ2022-35/Suizhou/2022	OQ388848		OQ389065		OQ388933	
HBSZ2021-28/Suizhou/2021	OQ388849	OQ388753	OQ389044	OQ389104	OQ388934	OQ388966
HBXG2022-09/Xiaogan Dawu/2022	OQ388850	OQ388754	OQ389079	OQ389124	OQ388935	OQ388968
HBSZ2022-10/Suizhou/2022	OQ388851	OQ388755	OQ389045	OQ389105	OQ388936	OQ388969
HBSZ2022-11/Suizhou/2022	OQ388852	OQ388756	OQ389046	OQ389106	OQ388937	OQ388970
HBHG2022-12/Huanggang_Qichun/2022	OQ388853	OQ388757	OQ389035	OQ389099	OQ388938	OQ388971
HBHG2022-13/Huanggang_Luotian/2022	OQ388854		OQ389036		OQ388940	
HBWH2022-14/Wuhan Xinzhou/2022	OQ388855		OQ389069		OQ388939	
HBHG2022-15/Huanggang_Hongan/2022	OQ388856		OQ389037		OQ388941	
HBHG2022-16/Huanggang_Yingshan/2022	OQ388857		OQ389038		OQ388942	
HBHG2022-36/Huanggang_Luotian/2022	OQ388863	OQ388758	OQ389039	OQ389100	OQ388943	OQ388973
HB2022-X37/Hubei/2022	OQ388862	OQ388765	OQ389000	OQ389081	OQ388944	OQ388976
HBHG2022-X39/Huanggang_Yingshan/2022	OQ388861	OQ388764	OQ389041	OQ389101	OQ388945	OQ388977
HBHG2022-X40/Huanggang_Macheng/2022	OQ388860	OQ388763	OQ389042	OQ389102	OQ388946	OQ388981
HBHG2022-X41/Huanggang_Yingshan/2022	OQ388859	OQ388762	OQ389043	OQ389103	OQ388947	OQ388978
HBYC2022-X49/Yichang/2022	OQ388858	OQ388761	OQ389080	OQ389125	OQ388948	OQ388979
HBHG2021-20/Huanggang Qichun/2021	OQ388867	OQ388777	OQ389025	OQ389096	OQ388952	OQ388974
HBSZ2022-54/Suizhou/2022	OQ388866	OQ388759	OQ389066	OQ389116	OQ388951	OQ388972
HBHG2022-52/Huanggang_Yingshan/2022	OQ388864		OQ389040		OQ388949	
HBWH2022-53/Wuhan_Xinzhou/2022	OQ388865		OQ389070		OQ388950	
HBHG2021-21/Huanggang_Yingshan/2021	OQ388868		OQ389026		OQ388953	
HBHG2021-06/Huanggang_Yingshan/2021	OQ388869		OQ389022		OQ388954	
