# Supplementary material for: Extensive genetic diversity of severe fever with thrombocytopenia syndrome virus circulating in Hubei Province, China, 2018–2022
Source: PLoS Negl Trop Dis. 2023 Sep 18;17(9):e0011654. doi: 10.1371/journal.pntd.0011654 (PMC10538666; doi:10.1371/journal.pntd.0011654)
Supplement: S3 Table — (PDF) [file pntd.0011654.s003.pdf]

S3 Table. SFTSV sequences of L, M and S available from GenBank were listed with name of isolate, location and accession number.

Name	Location	Accession No.		
		L	M	S
HB2014-36/Huanggang/2014	Hubei	KY933705	KY933693	KY933680
HB2016-080/Huanggang_Luotian/2016	Hubei	KY965123	KY965105	KY965088
HB2016-073/Huanggang_Luotian/2016	Hubei	KY965121	KY965103	KY965086
HB2016-034/Huanggang/2016	Hubei	KY965116	KY965098	KY965081
HB2014-22/Suizhou/2014	Hubei	KY933701	KY933688	KY933675
HB2014-13/Huanggang_xishui/2014	Hubei	KY933697	KY933684	KU738908
HB2017-17/Huanggang_Luotian/2017	Hubei	MK524359	MK524358	MK524357
HB2015-09/Huanggang_Luotian/2015	Hubei	MK513906	MK513905	MK513904
HB2014-31/Huanggang_Luotian/2014	Hubei	KY933707	KY933691	KY933678
HB2016-038/Huanggang_Yingshan/2016	Hubei	KY965117	KY965099	KY965082
HB2016-106/Hubei_Shiyan/2016	Hubei	KY965126	KY965108	KY965091
HB153/Hubei/2012	Hubei	MK355641	MK355640	KU664013.
HB2017-25/Huanggang_Macheng/2017	Hubei	MK524365	MK524364	MK524363
HB2016-013/Hubei_Xiaogan/2016	Hubei	KY965112	KY965094	KY965077
HB2014-20/Hubei_Wuhan/2014	Hubei	KY933699	KY933686	KU738910
HB123/Hubei/2011	Hubei	MK355635	MK355634	KU664010
HB2015-34/Huanggang_Hongan/2015	Hubei	MK513927	MK513926	MK513925
HBHG36/Hubei_Huanggang/2017	Hubei	MT320796	MT320797	MT320798
HB154/China/2011	China	JQ733561	JQ733560	JQ733562
HB2014-08/Suizhou_Guangshui/2014	Hubei	KU529304	KY933682	KU738905
HB2014-04/Huanggang_Hongan/2014	Hubei	KU524075	KR080475	KP984502
HB2015-23/Huanggang_Hongan/2015	Hubei	MK513915	MK513914	MK513913
HBHG29/Hubei/2017	Hubei	MT320787	MT320788	MT320789
HBHG30/Hubei/2017	Hubei	MT320790	MT320791	MT320792
HBHG35/Hubei/2017	Hubei	MT320793	MT320794	MT320795
HBHG38/Hubei/2017	Hubei	MT320799	MT320800	MT320801
HBXG51/Hubei/2017	Hubei	MT320802	MT320803	MT320804
HBSZ52/Hubei/2017	Hubei	MT320805	MT320806	MT320807
HBHG54/Hubei/2017	Hubei	MT320808	MT320809	MT320810
HBSZ55/Hubei/2017	Hubei	MT320811	MT320812	MT320813
HBHG8/Hubei/2017	Hubei	MT320814	MT320815	MT320816
HBSZ11/Hubei/2017	Hubei	MT320817	MT320818	MT320819
SFTSV/LTSL1/Hubei/2019	Hubei	MW721874	MW721876	MW721878
HB29/Hubei/2010	Hubei	KP202163	KP202164	KP202165
HB156/China/2011	Hubei	JQ733567	JQ733566	JQ733568
HB155/China/2011	Hubei	JQ733564	JQ733563	JQ733565
HB29/China/2010	Hubei	HM745930	HM745931	HM745932
HB3-sheep03/China/2011	Hubei	MK355655	MK355654	KU664009
HBMC16_human_2015/Hubei/2015	Hubei	KY440777	KY440776	KY440769
HBGS13_human_2015/Hubei/2015	Hubei	KY440774	KY440773	KY440772
HBMC5_human_2016/Hubei/2016	Hubei	KY440771	KY440770	KY440769
HB2012-196/Hubei/2012	Hubei	MT114241	MT114255	MT114285
HB2012-193/Hubei/2012	Hubei	MT114240	MT114254	MT114284
HB2016-003/Hubei/2016	Hubei	KY965109	KY965092	KY965074
HB2013-048/Hubei/2013	Hubei	MT114244	MT114258	MT114288

HB2012-197/Hubei/2012	Hubei	MT114242	MT114256	MT114286
HNXY_278/Henan_Xinyang/2014	Henan xinyang	KC292348	KC292322	KC292295
MN510013/Henan-277/Henan/2018	Henan	MN510013	MN510167	NA
SDLZtick12/China/2010	China	JQ684871	JQ684872	JQ684873
SPL003A/Japan/2012	Japan	AB817980	AB817988	AB817996
YG1/Japan/2012	Japan	AB817979	AB817987	AB817995
YGS1/Henan Xinyang/2011	Henan Xinyang	KF356549	KF356537	KF356525
Zhao/Zhoushan/2013	Zhoushan	KF374682	KF374684	KF374683
Zhejiang/01/Zhejiang Zhoushan/2011	Zhejiang Zhoushan	KJ597825	KJ597824	KJ597823.
ZJZHSH-LU/China/10/2011	zhoushan	KR017831	KR017850	KR017812
ZJZHSH-WRF/China/08/2014	Zhoushan	KR017845	KR017864	KR017826
ZJZHSH-WSH/China/08/2012	zhoushan	KR017835	KR017854	KR017816
2010-FQM/Henan/2011	Henan	HQ419227	HQ419236	HQ419240
2011YPQ11/Henan Xinyang/2011	Henan Xinyang	KF711886	KF711926	KF711897
AH12/Anhui/2010	Anhui	HQ116417	HQ141590	HQ141591
AHL/China Anhui/2011	Anhui	JQ670934	JQ670930	JQ670932
AHZ/China Anhui/2011	Anhui	JQ670929	JQ670931	JQ670933
BX-2010/Henan/2010	Hennan	JF682773	JF682774	JF682775
Gangwon/Korea/2012	Korea	KF358691	KF358692	KF358693
HL/Adult/G2/2013	China	KF791962	KF791957	KF791952
HL/Egg/G2/2013	China	KF791959	KF791954	KF791949
HL/Injected/2013	China	KF791958	KF791953	KF791948
HL/Larvae/G2/2013	China	KF791960	KF791955	KF791950
HL/Nymph/G2/2013	China	KF791961	KF791956	KF791951
HNXY_212/Henan/2013	Henan	KC292327	KC292300	KC292273
HNXY_293/Henan/2013	Henan Xinyang	KC292330	KC292303	KC292276
JS4/China/2010	Jiangsu	HQ141604	HQ141605	HQ141606
JS2012-tick01/Jiangsu/2012	Jiangsu	KC473540	KC473541	KC473542
KAGWH3/Korea/2014	Korea	KP663734	KP663735	KP663736
SD4/China/2010	China	HM802202	HM802203	HM802204
LN2012-58/Liaoning/2012	Liaoning	KF887445	KF887440	KF887435
LN2012-42/Liaoning/2012	Liaoning	KF887444	KF887439	KF887434
LN2012-41/Liaoning/2012	Liaoning	KF887443	KF887438	KF887433
LN2012-34/Liaoning/2012	Liaoning	KF887442	KF887437	KF887432
LN2012-14/Liaoning/2012	Liaoning	KF887441	KF887436	KF887431
LN2013-030/Liaoning/2013	Liaoning	MT114212	MT114228	MT114272
LN2011-037/Liaoning/2011	Liaoning	MT114203	MT114219	MT114263
LN2011-xq/Liaoning/2011	Liaoning	MT114205	MT114220	MT114264
LN2012-049/Liaoning/2012	Liaoning	MT114209	MT114225	MT114269
LN2013-047/Liaoning/2013	Liaoning	MT114214	MT114230	MT114274
LN2/China/2010	Liaoning	HQ141607	HQ141608	HQ141609
LN3/China/2010	Liaoning	HQ141610	HQ141611	HQ141612
SD24/China/2010	Shandong	HM802200	HM802201	HM802205
SDTA_4/Shandong/2015	Shandong	KX641912	KX641916	KX641920
SDTA_3/Shandong/2015	Shandong	KX641911	KX641915	KX641919
SDTA_2/Shandong/2015	Shandong	KX641910	KX641914	KX641918
SDTA_1/Shandong/2015	Shandong	KX641909	KX641913	KX641917
SD2011-040/Shandong/2011	Shandong	MT005209	MT005251	MT114299
SD2014-157/Shandong/2014	Shandong	MT005233	MT005275	MT114323
SD2014-163/Shandong/2014	Shandong	MT005234	MT005276	MT114324
SD2013-058/Shandong/2013	Shandong	MT005221	MT005263	MT114311

SD2011-028/Shandong/2011	Shandong	MT005202	MT005244	MT114292
SD2012-076/Shandong/2012	Shandong	MT005216	MT005258	MT114306
SD2013-076/Shandong/2013	Shandong	MT005223	MT005265	MT114313
SD2013-080/Shandong/2013	Shandong	MT005225	MT005267	MT114315
SD2011-038/Shandong/2011	Shandong	MT005207	MT005249	MT114297
SD2014-209/Shandong/2014	Shandong	MT005236	MT005278	MT114326
JS2014-Hedgehog-02/Jiangsu/2014	Jiangsu	KR230769	KR230789	KR230809
JS2014-Hedgehog-01/Jiangsu/2014	Jiangsu	KR230768	KR230788	KR230808
JS2014-H.longicornis-03/Jiangsu/2014	Jiangsu	KR230767	KR230787	KR230807
JS2014-H.longicornis-02/Jiangsu/2014	Jiangsu	KR230766	KR230786	KR230806
JS2014-H.longicornis-01/Jiangsu/2014	Jiangsu	KR230765	KR230785	KR230805
JS2014-39//Jiangsu/2014	Jiangsu	KR230764	KR230784	KR230804
JS2014-33/Jiangsu/2014	Jiangsu	KR230763	KR230783	KR230803
JS2014-31/Jiangsu/2014	Jiangsu	KR230762	KR230782	KR230802
JS2014-18/Jiangsu/2014	Jiangsu	KR230761	KR230781	KR230801
JS2015-32/Jiangsu/2015	Jiangsu	KY362317	KY362365	KY362341
JS2014-16/Jiangsu/2014	Jiangsu	KR230760	KR230780	KR230800
JS2014-15/Jiangsu/2014	Jiangsu	KR230759	KR230779	KR230799
JS2014-03/Jiangsu/2014	Jiangsu	KR230758	KR230778	KR230798
JS2013-71/Jiangsu/2013	Jiangsu	KR230757	KR230777	KR230797
JS2013-69/Jiangsu/2013	Jiangsu	KR230756	KR230776	KR230796
JS2013-52/Jiangsu/2013	Jiangsu	KR230755	KR230775	KR230795
JS2013-46/Jiangsu/2013	Jiangsu	KR230754	KR230774	KR230794
JS2013-44/Jiangsu/2013	Jiangsu	KR230753	KR230773	KR230793
JS2013-31/Jiangsu/2013	Jiangsu	KY362303	KY362351	KY362327
JS2015-01/Jiangsu/2015	Jiangsu	KY362316	KY362364	KY362340
JS2012-79/Jiangsu/2012	Jiangsu	KR230752	KR230772	KR230792
JS2011-062/Jiangsu/2011	Jiangsu	KC505135	KC505136	KC505137
JS2011-69/Jiangsu/2011	Jiangsu	KR230751	KR230771	KR230791
JS2011-13-2/Jiangsu/2011	Jiangsu	KR230750	KR230770	KR230790
JS2011-013-1/Jiangsu/2011	Jiangsu	KC505126	KC505127	KC505128
JS2010-019/Jiangsu/2010	Jiangsu	JQ317178	JQ317179	JQ317180
JS2010-015/Jiangsu/2010	Jiangsu	JQ317172	JQ317173	JQ317174
JSD1/Jiangsu/2011	Jiangsu	JF267783	JF267784	JF267785
JS2011-034/Jiangsu/2011	Jiangsu	KC505132	KC505133	KC505134
JS2010-018/Jiangsu/2010	Jiangsu	JQ317175	JQ317176	JQ317177
JS2010-014/Jiangsu/2010	Jiangsu	JQ317169	JQ317170	JQ317171
JS2012-goat01/Jiangsu/2012	Jiangsu	KC473537	KC473538	KC473539
JS2011-027/Jiangsu/2011	Jiangsu	KC505129	KC505130	KC505131
JS6/Jiangsu/2010	Jiangsu	HQ830169	HQ830170	HQ830171
JS3/China/2010	Jiangsu	HQ141601	HQ141602	HQ141603
JS2012-020/Jiangsu/2012	Jiangsu	KC505141	KC505142	KC505143
JS26/Jiangsu/2010	Jiangsu	HQ830166	HQ830167	HQ830168
JS24/Jiangsu/2010	Jiangsu	HQ830163	HQ830164	HQ830165
JS2011-004/Jiangsu/2011	Jiangsu	KC505123	KC505124	KC505125
JS2007-01/Jiangsu/2011	Jiangsu	JF837593	JF837594	JF837595
JS2012-035/Jiangsu/2012	Jiangsu	KC505144	KC505145	KC505146
JS2011-109/Jiangsu/2012	Jiangsu	KC505138	KC505139	KC505140
SPL097A/Japan/2015	Japan	AB983518	AB985312	AB985544
SPL100A/Japan/2015	Japan	AB983519	AB985313	AB985545

SPL057A/Japan/2015	Japan	AB983500	AB985295	AB985526
SPL004A/Japan/2014	Japan	AB817981	AB817989	AB817997
SPL073A/Japan/2015	Japan	AB985637	NA	NA
SPL077A/Japan/2016	Japan	AB983510	AB985304	AB985536
SPL084A/Japan/2016	Japan	AB983513	AB985307	AB985539
SPL067A/Japan/2016	Japan	AB983504	AB985299	AB985530
SPL082A/Japan/2016	Japan	AB983512	AB985306	AB985538
SPL090A/Japan/2016	Japan	AB983517	AB985311	AB985543
SPL062A/Japan/2016	Japan	AB983502	AB985297	AB985528
SPL078A/Japan/2016	Japan	AB983511	AB985305	AB985537
SPL112A/Japan/2016	Japan	AB983525	AB985319	AB985551
SPL086A/Japan/2016	Japan	AB983514	AB985308	AB985540
SPL075A/Japan/2016	Japan	AB983509	AB985303	AB985535
SPL108A/Japan/2016	Japan	AB983524	AB985318	AB985550
SPL053A/Japan/2016	Japan	AB983497	AB985292	AB985523
SPL128A/Japan/2016	Japan	AB983532	AB985325	AB985558
SPL104A/Japan/2016	Japan	AB983520	AB985314	AB985546
SPL055A/Japan/2016	Japan	AB983499	AB985294	AB985525
SPL089A/Japan/2016	Japan	AB983516	AB985310	AB985542
SPL035A/Japan/2014	Japan	AB817986	AB817994	AB818002
SPL069A/Japan/2016	Japan	AB983505	AB985300	AB985531
SPL105A/Japan/2016	Japan	AB983521	AB985315	AB985547
SPL010A/Japan/2014	Japan	AB817983	AB817991	AB817999
SPL060A/Japan/2016	Japan	AB983501	AB985296	AB985527
SPL071A/Japan/2016	Japan	AB983507	AB985302	AB985533
SPL125A/Japan/2016	Japan	AB983531	NA	AB985557
SPL030A/Japan/2014	Japan	AB817984	AB817992	AB818000
SPL005A/Japan/2014	Japan	AB817982	AB817990	AB817998
SPL032A/Japan/2014	Japan	AB817985	AB817993	AB818001
SPL124A/Japan/2016	Japan	AB983530	AB985324	AB985556
SPL121A/Japan/2016	Japan	AB983529	AB985323	AB985555
SPL120A/Japan/2016	Japan	AB983528	AB985322	AB985554
SPL117A/Japan/2016	Japan	AB983527	AB985321	AB985553
SPL114A/Japan/2016	Japan	AB983526	AB985320	AB985552
SPL107A/Japan/2016	Japan	AB983523	AB985317	AB985549
SPL106A/Japan/2016	Japan	AB983522	AB985316	AB985548
SPL129A/Japan/2016	Japan	AB983533	AB985326	AB983533
SPL161A/Japan/2014	Japan	AB983534	NA	AB985572
SPL070A/Japan/2016	Japan	AB983506	AB985301	AB985532
SPL072A/Japan/2016	Japan	AB983508	NA	AB985534
SPL054A/Japan/2016	Japan	AB983498	AB985293	AB985524
SPL066A/Japan/2016	Japan	AB983503	AB985298	AB985529
KACNH3/Korea/2015	South Korea	KP663743	KP663744	KP663745
KAGBH6/Korea/2015	South Korea	KP663740	KP663741	KP663742
KAGBH5/Korea/2015	South Korea	KP663737	KP663738	KP663739
KASJH/Korea/2014	South Korea	KP663731	KP663732	KP663733
CB1/Korea/2014	South Korea	KY789433	KY789436	KY789439
14KS52/Korea/2014	South Korea	MG737007	MG737116	MG737224
16KS55/Korea/2016	South Korea	MG737046	MG737155	MG737263
15MS31/Korea/2015	South Korea	MG737018	MG737127	MG737235
2015SH239S/Henan/2015	Henan	NA	NA	KT736099

YSH147/Henan/2015	Henan	NA	NA	KT721308
YSH127/Henan/2015	Henan	NA	NA	KT721307
12-China_Henan-100	Henan	NA	NA	MN510193
2013048S/Henan Xinyang/2013	Henan	NA	NA	KF917432
2015SYSH86/Henan/2015	Henan	NA	NA	KT380657
2015SH171S/Henan/2015	Henan	NA	NA	KT736102
HNXY_31/Henan Xinyang/2013	Henan	KC292336	KC292310	KC292283
HNXY_206/Henan Xinyang/2013	Henan	KC292329	KC292302	KC292275
HNXY2017-50/Henan Xinyang/2017	Henan	NA	NA	MN147875
18-China_Henan-327/Henan/2018	Henan	NA	MN510171	MN510322
2012YSH91/Henan Xinyang/2012	Henan	KF711883	KF711920	KF711891
YSHX002/Henan Xinyang/2014	Henan	KF356551	KF356540	KF356528
2012YSH6/Henan Xinyang/2012	Henan	KF711882	KF711921	KF711892
2012YSH9/Henan Xinyang/2012	Henan	KF711881	KF711919	KF711890
HNXY_327/Henan Xinyang/2013	Henan	KC292347	KC292321	KC292294
2011YXX9/Henan Xinyang/2011	Henan	KF711889	KF711924	KF711898
2011YGS5/Henan Xinyang/2011	Henan	KF711884	KF711925	KF711893
2011YPQ17/Henan Xinyang/2011	Henan	KF711887	KF711923	KF711896
2011YSC22/Henan Xinyang/2011	Henan	KF711888	KF711927	KF711894
2011YGS7/Henan Xinyang/2011	Henan	KF711885	KF711922	KF711895
YSC19/Henan Xinyang/2011	Henan	KF356548	KF356536	KF356524
2011YPQ12/Henan Xinyang/2011	Henan	KF711880	KF711930	KF711915
HNXY_115/Henan Xinyang/2013	Henan	KC292338	KC292312	KC292285
HNXY_93/Henan Xinyang/2013	Henan	KC292337	KC292311	KC292284
YSH39/Henan Xinyang/2011	Henan	KF356541	KF356529	KF356517
HN6/China/2010	Henan	HQ141595	HQ141596	HQ141597
YSC3/Henan Xinyang/2011	Henan	KF356547	KF356535	KF356523
HNXY_231/Henan Xinyang/2013	Henan	KC292339	KC292313	KC292286
HNXY_207/Henan Xinyang/2013	Henan	KC292341	KC292315	KC292288
2012YGS10/Henan Xinyang/2012	Henan	KF711872	KF711937	KF711909
2012YSH14/Henan Xinyang/2012	Henan	KF711876	KF711938	KF711910
2012YSH86/Henan Xinyang/2012	Henan	KF711877	KF711939	KF711907
2012YSH37/Henan Xinyang/2012	Henan	KF711871	KF711936	KF711905
2012YSH16/Henan Xinyang/2012	Henan	KF711874	KF711934	KF711908
HNXY_2/Henan Xinyang/2013	Henan	KC292344	KC292318	KC292291
2012YSH89/Henan Xinyang/2012	Henan	KF711873	KF711935	KF711906
HNXY_319/Henan Xinyang/2013	Henan	KC292345	KC292319	KC292292
HNXY_164/Henan Xinyang/2013	Henan	KC292340	KC292314	KC292287
2012YSC6/Henan Xinyang/2012	Henan	KF711879	KF711931	KF711902
2012YSH27/Henan Xinyang/2012	Henan	KF711878	KF711932	KF711903
HNXY_130/Henan Xinyang/2013	Henan	KC292342	KC292316	KC292289
HNXY_195/Henan Xinyang/2013	Henan	KC292352	KC292326	KC292299
HNXY_224/Henan Xinyang/2013	Henan	KC292343	KC292317	KC292290
HN-LR/China/05/2012	Henan	KR017842	KR017861	KR017823
2012YGS4/Henan Xinyang/2012	Henan	KF711875	KF711933	KF711904
YXX1/Henan Xinyang/2011	Henan	KF356545	KF356533	KF356521
YPQ2/Henan Xinyang/2011	Henan	KF356543	KF356531	KF356519
2011YSC45/Henan Xinyang/2011	Henan	KF711870	NA	NA
YNY1/Henan Xinyang/2011	Henan	KF356552	KF356538	KF356526
HNXY_144/Henan Xinyang/2013	Henan	KC292333	KC292307	KC292280
YPQ133/Henan Xinyang/2011	Henan	KF356542	KF356530	KF356518

HNXY_182/Henan Xinyang/2013	Henan	KC292350	KC292324	KC292297
HNXY_186/Henan Xinyang/2013	Henan	KC292334	KC292308	KC292281
YPQ5/Henan Xinyang/2011	Henan	KF356544	KF356532	KF356520
HNXY_262/Henan Xinyang/2013	Henan	KC292335	KC292309	KC292282
HNXY_174/Henan Xinyang/2013	Henan	KC292332	KC292306	KC292279
BX-2010-01/Henan/2010	Henan	HQ642766	HQ642767	HQ642768
BX-2010-69/Henan/2010	Henan	JF682776	JF682777	JF682778
HNXY_245/Henan Xinyang/2013	Henan	KC292346	KC292320	KC292293
2012YSH105/Henan Xinyang/2012	Henan	KF711869	KF711944	KF711914
HNXY_202/Henan Xinyang/2013	Henan	KC292351	KC292325	KC292298
2012YSH104/Henan Xinyang/2012	Henan	KF711867	KF711943	KF711912
2012YSH93/Henan Xinyang/2012	Henan	KF711866	KF711946	KF711913
YPQX03/Henan Xinyang/2011	Henan	KF356550	KF356539	KF356527
2012YSH10/Henan Xinyang/2012	Henan	KF711868	KF711940	KF711911
HNXY_157/Henan Xinyang/2013	Henan	KC292331	KC292305	KC292278
HNXY_170/Henan Xinyang/2013	Henan	KC292353	KC292304	KC292277
2012YSH92/Henan Xinyang/2012	Henan	KF711865	KF711941	KF711917
2012YSH107/Henan Xinyang/2012	Henan	KF711864	KF711942	KF711916
HNXY_191/Henan Xinyang/2013	Henan	KC292349	KC292323	KC292296
2011YSC60/Henan Xinyang/2011	Henan	KF711863	KF711945	KF711899
YXX2/Henan Xinyang/2011	Henan	KF356546	KF356534	KF356522
HN13/China/2010	Henan	HQ141598	HQ141599	HQ141600
2011YSC52/Henan Xinyang/2011	Henan	KF711861	NA	NA
HNXY_188/Henan Xinyang/2013	Henan	KC292328	KC292301	KC292274
2012YXX1/Henan Xinyang/2012	Henan	KF711862	KF711929	KF711901
2011YSH52/Henan Xinyang/2011	Henan	NA	KF711928	KF711900
ZJ2013-06/Zhejiang/2013	Zhejiang	KP280206	KP280204	KP280205
ZJZHSH-CXD/China/05/2012	Zhejiang	KR017839	KR017858	KR017820
ZJZHSH-FZHR/China/07/2013	Zhejiang	KR017843	KR017862	KR017824
ZL13-32/Zhejiang/2013	Zhejiang	KU361343	KU361342	KU361341
ZJ2018-01/Zhejiang/2018	Zhejiang	MZ561692	MZ561691	MZ561690
LS19-02/Zhejiang/2019	Zhejiang	NA	NA	MN480460
ZJZHSH-YJX/China/06/2012	Zhejiang	KR017841	KR017860	KR017822
ZJZHSH-AM/China/05/2012	Zhejiang	KR017827	KR017846	KR017808
ZJZHSH-LWL/China/08/2014	Zhejiang	KR017844	KR017863	KR017825
ZJZHSH-XAM/China/06/2012	Zhejiang	KR017834	KR017853	KR017815
ZJZHSH-HYC/China/05/2012	Zhejiang	KR017828	KR017847	KR017809
ZJZHSH-SHZ/China/05/2012	Zhejiang	KR017829	KR017848	KR017810
ZJZHSH-HYJ/China/07/2012	Zhejiang	KR017833	KR017852	KR017814
ZJZHSH-FDE/China/06/2012	Zhejiang	KR017840	KR017859	KR017821
ZJZHSH-ZLN/China/06/2012	Zhejiang	KR017837	KR017856	KR017818
ZJZHSH-LHZH/China/06/2012	Zhejiang	KR017838	KR017857	KR017819
ZJZHSH-HCY/China/06/2012	Zhejiang	KR017836	KR017855	KR017817
ZJZHSH-ZLD/China/06/2012	Zhejiang	KR017832	KR017851	KR017813
AH15/China/2010	Anhui	HQ141592	HQ141593	HQ141594
AH-YTY/China/05/2012	Anhui	KR017830	KR017849	KR017811
JX2013-01/Jiangxi/2013	Jiangxi	MN245796	MN245797	MN245798
SFTSV_JX2013/Jiangxi/2013	Jiangxi	KY848825	KY848826	KY848827
TP1910a/Taiwan/2019	Taiwan	MN910270	MN830174	MN830173
16MS373/Korea/2016	South Korea	MG737073	MG737182	MG737290
SPL087A/Japan/2014	Japan	AB983515	AB985309	AB985541

DS03/CHN/2013	China	KR698346	KR698333	KR698320
DS05/CHN/2013	China	KR698348	KR698335	KR698322
DS04/CHN/2013	China	KR698347	KR698334	KR698321
DS02/CHN/2013	China	KR698345	KR698332	KR698319
2010-WSQ/China/2011	China	HQ419226	HQ419235	HQ419239
JN1/China/2010	China	JN258707	JN258706	JN258705
HZM/China/2010	China	HQ171188	JF951393	HQ171193
WWG/China/2010	China	HQ171189	HQ419231	HQ171194
QD7/China/2014	China	KR706567	KR706566	KR706565
HGX/China/2010	China	HQ171187	NA	NA
WJ/China/2009	China	HQ171186	HQ419229	HQ171191
Huaiyangshan virus/China/2010	China	JF906056	JF906057	NA
2010-T112/China/2010	China	HQ419228	HQ419238	NA
NB34/CHN/2013	China	KR698353	KR698340	KR698327
NB08/CHN/2013	China	KR698349	KR698336	KR698323
NB38/CHN/2013	China	KR698354	KR698341	KR698328
HNXH/China/2014	China	KT254588	NC_043451	KT254589
WJQ/China/2010	China	HQ171190	HQ419230	HQ171195
NB31/CHN/2013	China	KR698351	KR698338	KR698325
NB32/CHN/2013	China	KR698352	KR698339	KR698326
NB39/CHN/2014	China	KR698355	KR698342	KR698329
NB24/CHN/2013	China	KR698350	KR698337	KR698324
Rodent02/NB/CHN/2014	China	KR698357	KR698344	KR698331
Rodent01/NB/CHN/2014	China	KR698356	KR698343	KR698330
KR 1020150042419-A/1	NA	DI493114	DI493115	NA
KR 1020150042419-A/3	NA	NA	NA	DI493116
CP01/Korea/2013	Korea	NA	NA	KR612073
SPL169A/Japan/2014	Japan	NA	NA	AB985575
SPL148A/Japan/2014	Japan	NA	NA	AB985568
SPL136A/Japan/2014	Japan	NA	NA	AB985563
SPL172A/Japan/2014	Japan	NA	NA	AB985578
SPL163A/Japan/2014	Japan	NA	NA	AB985573
SPL132A/Japan/2014	Japan	NA	NA	AB985561
SPL137A/Japan/2014	Japan	NA	NA	AB985564
SPL134A/Japan/2014	Japan	NA	NA	AB985562
SPL168A/Japan/2014	Japan	NA	NA	AB985574
SPL142A/Japan/2014	Japan	NA	NA	AB985566
SPL141A/Japan/2014	Japan	NA	NA	AB985565
SPL130A/Japan/2014	Japan	NA	NA	AB985560
SPL146A/Japan/2014	Japan	NA	NA	AB985567
AP01/Korea/2013	Korea	NA	NA	KR612072
SPL171A/Japan/2014	Japan	NA	NA	AB985577
DP01/Korea/2013	Korea	NA	NA	KR612074
SPL160A/Japan/2014	Japan	NA	NA	AB985571
SPL154A/Japan/2014	Japan	NA	NA	AB985569
SPL170A/Japan/2014	Japan	NA	NA	AB985576
SPL158A/Japan/2014	Japan	NA	NA	AB985570
JP02/Korea/2013	Korea	NA	NA	KR612078
JP01/Korea/2013	Korea	NA	NA	KR612076
JP07/Korea/2013	Korea	NA	NA	KR612088
JP03/Korea/2013	Korea	NA	NA	KR612079

JP01/Korea/2014	Korea	NA	NA	KR612075
JP06/Korea/2014	Korea	NA	NA	KR612085
JP03/Korea/2014	Korea	NA	NA	KR612080
JP06/Korea/2013	Korea	NA	NA	KR612086
JP02/Korea/2014	Korea	NA	NA	KR612077
JP04/Korea/2014	Korea	NA	NA	KR612082
JP07/Korea/2014	Korea	NA	NA	KR612087
JP05/Korea/2014	Korea	NA	NA	KR612084
JP05/Korea/2013	Korea	NA	NA	KR612083
JP04/Korea/2013	Korea	NA	NA	KR612081
SDLZP07/China/2011	China	NA	NA	JQ693010
SDLZP08/China/2011	China	NA	NA	JQ693011
YSH96/Henan/2015	Henan	NA	NA	KT721300
2012YSH45/Henan Xinyang/2012	Henan Xinyang	NA	NA	KF711918
2013063S/Henan Xinyang/2013	Henan Xinyang	NA	NA	KF917444
HB2014-15/Shiyan_Maojian/2014	Shiyan_Maojian	NA	NA	KU738909
2013079S/Henan_Xinyang/2013	Henan Xinyang	NA	NA	KF917442
2013077S/Henan_Xinyang/2013	Henan Xinyang	NA	NA	KF917441
2013052S/Henan_Xinyang/2013	Henan Xinyang	NA	NA	KF917443
48S//Henan_Xinyang/2014	Henan Xinyang	NA	NA	KP339885
HB2014-10/Shuizhou_Zhengdu/2014	huizhou_Zhengdu	NA	NA	KU738906
2015SNiB1/Henan/2015	Henan	NA	NA	KT380655
2014187S/Henan/2014	Henan	NA	NA	KR075915
2015SH224S/Henan/2015	Henan	NA	NA	KT736095
2013081S/Henan_Xinyang/2013	Henan Xinyang	NA	NA	KF917438
149S/Henan/2014	Henan	NA	NA	KP339920
144S/Henan/2014	Henan	NA	NA	KP339918
2013001S/Henan_Xinyang/2013	Henan Xinyang	NA	NA	KF917439
2015SH221S/Henan/2015	Henan	NA	NA	KT736094
YSH60/Henan/2015	Henan	NA	NA	KT721319
2015SYSH33/Henan/2015	Henan	NA	NA	KT380653
2015SYPQ26/Henan/2015	Henan	NA	NA	KT380645
2015SYPQ3/Henan/2015	Henan	NA	NA	KT380644
17S/Henan/2014	Henan	NA	NA	KP339931
39S/Henan/2014	Henan	NA	NA	KP339940
105S/Henan/2014	Henan	NA	NA	KP339902
6S/Henan/2014	Henan	NA	NA	KP339927
156S/Henan/2014	Henan	NA	NA	KP339923
2013045S/Henan_Xinyang/2013	Henan Xinyang	NA	NA	KF917440
YSH107/Henan/2015	Henan	NA	NA	KT721301
2014186S/Henan/2014	Henan	NA	NA	KR075914
XCQ-A112S/China/2010	China	NA	NA	JF906058
HB2014-21/Xianning_Tongshan/2014	ianning_Tongsha	NA	NA	KU738911
SDLZP02/China/2011	China	NA	NA	JQ693005
SDLZP05/China/2011	China	NA	NA	JQ693008
SDLZP09/China/2011	China	NA	NA	JQ693012
SDLZP06/China/2011	China	NA	NA	JQ693009
SDLZP04/China/2011	China	NA	NA	JQ693007
SDLZP03/China/2011	China	NA	NA	JQ693006
YSH130/Henan/2015	Henan	NA	NA	KT721313
2014221S/Henan/2014	Henan	NA	NA	KR075913

127S/Henan/2014	Henan	NA	NA	KP339909
65S/Henan/2014	Henan	NA	NA	KP339889
125S/Henan/2014	Henan	NA	NA	KP339908
116S/Henan/2014	Henan	NA	NA	KP339905
81S/Henan/2014	Henan	NA	NA	KP339895
YSH166/Henan/2015	Henan	NA	NA	KT721317
YSH139/Henan/2015	Henan	NA	NA	KT721314
YSH165/Henan/2015	Henan	NA	NA	KT721318
YSH161/Henan/2015	Henan	NA	NA	KT721316
YSH159/Henan/2015	Henan	NA	NA	KT721315
YSH162/Henan/2015	Henan	NA	NA	KT721311
YSH138/Henan/2015	Henan	NA	NA	KT721299
YSH137/Henan/2015	Henan	NA	NA	KT721296
34S/Henan/2014	Henan	NA	NA	KP339938
67S/Henan/2014	Henan	NA	NA	KP339890
28S/Henan/2014	Henan	NA	NA	KP339934
2015PQ36S/Henan/2015	Henan	NA	NA	KT736090
YSH122/Henan/2015	Henan	NA	NA	KT721295
2013041S/Henan_Xinyang/2013	Henan_Xinyang	NA	NA	KF917430
2013066S/Henan_Xinyang/2013	Henan_Xinyang	NA	NA	KF917428
82s/Henan/2014	Henan	NA	NA	KP339896
YSH101/Henan/2015	Henan	NA	NA	KT721297
2015SYSH41/Henan/2015	Henan	NA	NA	KT380654
9S/Henan/2014	Henan	NA	NA	KP339928
154S/Henan/2014	Henan	NA	NA	KP339922
2013040S/Henan_Xinyang/2013	Henan_Xinyang	NA	NA	KF917434
2013037S/Henan_Xinyang/2013	Henan_Xinyang	NA	NA	KF917437
HB2014-12/Huanggang_Hongan/2014	luanggang_Honga	NA	NA	KU738907
2010-WWX/China/2010	China	NA	NA	HQ419242
YSH152/Henan/2015	Henan	NA	NA	KT721312
YSH145/Henan/2015	Henan	NA	NA	KT721306
2015SYSH30/Henan/2015	Henan	NA	NA	KT380652
151S/Henan/2014	Henan	NA	NA	KP339921
111S/Henan/2014	Henan	NA	NA	KP339904
98S/Henan/2014	Henan	NA	NA	KP339901
79S/Henan/2014	Henan	NA	NA	KP339894
2013075S/Henan_Xinyang/2013	Henan_Xinyang	NA	NA	KF917433
2013067S/Henan_Xinyang/2013	Henan_Xinyang	NA	NA	KF917429
2015PQ42S/Henan/2015	Henan	NA	NA	KT736091
2013051S/Henan_Xinyang/2013	Henan_Xinyang	NA	NA	KF917436
2013049S/Henan_Xinyang/2013	Henan_Xinyang	NA	NA	KF917435
2015SH242S/Henan/2015	Henan	NA	NA	KT736100
YSH136/Henan/2015	Henan	NA	NA	KT721310
2013043S/Henan_Xinyang/2013	Henan_Xinyang	NA	NA	KF917431
2015SYSH73/Henan/2015	Henan	NA	NA	KT380659
2015SH220S/Henan/2015	Henan	NA	NA	KT736093
129S/Henan/2014	Henan	NA	NA	KP339910
62S/Henan/2014	Henan	NA	NA	KP339888
YSH123/Henan/2015	Henan	NA	NA	KT721304
33S/Henan/2014	Henan	NA	NA	KP339937
18S/Henan/2014	Henan	NA	NA	KP339932

16S/Henan/2014	Henan	NA	NA	KP339930
12S/Henan/2014	Henan	NA	NA	KP339929
YSH113/Henan/2015	Henan	NA	NA	KT721298
2015SYSH5/Henan/2015	Henan	NA	NA	KT380648
61S/Henan/2014	Henan	NA	NA	KP339887
95S/Henan/2014	Henan	NA	NA	KP339899
2014101S/Henan/2014	Henan	NA	NA	KR075919
2015SYSH82/Henan/2015	Henan	NA	NA	KT380658
2015SYSH10/Henan/2015	Henan	NA	NA	KT380649
121S/Henan/2014	Henan	NA	NA	KP339906
2015SH246S/Henan/2015	Henan	NA	NA	KT736101
YSH126/Henan/2015	Henan	NA	NA	KT721305
21S/Henan/2014	Henan	NA	NA	KP339933
123S/Henan/2014	Henan	NA	NA	KP339907
YSH143/Henan/2015	Henan	NA	NA	KT721309
2013082S/Henan_Xinyang/2013	Henan_Xinyang	NA	NA	KF917427
2015SYSH25/Henan/2015	Henan	NA	NA	KT380651
93S/Henan/2014	Henan	NA	NA	KP339898
YSH98/Henan/2015	Henan	NA	NA	KT721294
YSH119/Henan/2015	Henan	NA	NA	KT721293
2015SYSH16/Henan/2015	Henan	NA	NA	KT380650
2015SGS5/Henan/2015	Henan	NA	NA	KT380646
41S/Henan/2014	Henan	NA	NA	KP339941
53S/Henan/2014	Henan	NA	NA	KP339886
2014181S/Henan/2014	Henan	NA	NA	KR075922
2014178S/Henan/2014	Henan	NA	NA	KR075921
2014175S/Henan/2014	Henan	NA	NA	KR075920
SDLZSheep01/China/2011	China	NA	NA	JQ693002
SDLZP01/China/2011	China	NA	NA	JQ693004
SDPLP01/China/2011	China	NA	NA	JQ693013
SDLZDog01/China/2011	China	NA	NA	JQ693003
SDLZCattle01/China/2011	China	NA	NA	JQ693001
2013050S/Henan_Xinyang/2013	Henan_Xinyang	NA	NA	KF917448
142S/Henan/2014	Henan	NA	NA	KP339916
164S/Henan/2014	Henan	NA	NA	KP339925
2015SH230S/Henan/2015	Henan	NA	NA	KT736096
87S/Henan/2014	Henan	NA	NA	KP339897
YSH11/Henan/2015	Henan	NA	NA	KT721320
2013039S/Henan_Xinyang/2013	Henan_Xinyang	NA	NA	KF917447
72S/Henan/2014	Henan	NA	NA	KP339891
2015SYSH84/Henan/2015	Henan	NA	NA	KT380656
2015SH232S/Henan/2015	Henan	NA	NA	KT736097
157S/Henan/2014	Henan	NA	NA	KP339924
2013038S/Henan_Xinyang/2013	Henan_Xinyang	NA	NA	KF917445
2015SH218S/Henan/2015	Henan	NA	NA	KT736092
135S/Henan/2014	Henan	NA	NA	KP339915
2014083S/Henan/2014	Henan	NA	NA	KR075924
2014045S/Henan/2014	Henan	NA	NA	KR075923
132S/Henan/2014	Henan	NA	NA	KP339912
38S/Henan/2014	Henan	NA	NA	KP339939
133S/Henan/2014	Henan	NA	NA	KP339913

134S/Henan/2014	Henan	NA	NA	KP339914
2015SGS21/Henan/2015	Henan	NA	NA	KT380647
74S/Henan/2014	Henan	NA	NA	KP339892
YSH111/Henan/2015	Henan	NA	NA	KT721302
131S/Henan/2014	Henan	NA	NA	KP339911
30S/Henan/2014	Henan	NA	NA	KP339935
2014197S/Henan/2014	Henan	NA	NA	KR075910
2013070S/Henan_Xinyang/2013	Henan_Xinyang	NA	NA	KF917446
2015SH233S/Henan/2015	Henan	NA	NA	KT736098
44S/Henan/2014	Henan	NA	NA	KP339884
143S/Henan/2014	Henan	NA	NA	KP339917
2010-CBX/China/2010	Henan	NA	NA	HQ419244
YSH116/Henan/2015	Henan	NA	NA	KT721303
165S/Henan/2014	Henan	NA	NA	KP339926
77S/Henan/2014	Henan	NA	NA	KP339893
2014218S/Henan/2014	Henan	NA	NA	KR075912
109S/Henan/2014	Henan	NA	NA	KP339903
2010-ZGQ/China/2010	Henan	NA	NA	HQ419241
2014203S/Henan/2014	Henan	NA	NA	KR075911
2014206S/Henan/2014	Henan	NA	NA	KR075909
2014073S/Henan/2014	Henan	NA	NA	KR075918
LZR/China/2010	Henan	NA	NA	HQ419243
147S/Henan/2014	Henan	NA	NA	KP339919
96S/Henan/2014	Henan	NA	NA	KP339900
2014196S/Henan/2014	Henan	NA	NA	KR075917
2014193S/Henan/2014	Henan	NA	NA	KR075916
32S/Henan/2014	Henan	NA	NA	KP339936
