# Supplementary material for: Extensive genetic diversity of severe fever with thrombocytopenia syndrome virus circulating in Hubei Province, China, 2018–2022
Source: PLoS Negl Trop Dis. 2023 Sep 18;17(9):e0011654. doi: 10.1371/journal.pntd.0011654 (PMC10538666; doi:10.1371/journal.pntd.0011654)
Supplement: S4 Table — (PDF) [file pntd.0011654.s004.pdf]

S4 Table. Association of all demographic, clinical variables and clinical manifestations with fatal case in the patients evaluated with the use of logistic regression model by adjusting sex and delay from onset to admission.

	Total	Fatal patients	Survival patients	Adjusted OR (95% CI)	*p* value
**Sex [no. (%)]**					
Male	103 (52.0)	21 (58.3)	82 (50.6)	1.366 (0.658-2.836)	0.403
Female	95 (48.0)	15 (41.7)	80 (49.4)		
**Age,y**					
Mean±SD	64.69±8.31	65.83±7.10	64.44±8.55	1.021 (0.976-1.069)	0.362
**Delay from onset to admission**					
Median (IQR)	7 (5-8)	7 (5-7.25)	7 (5-8)	0.971 (0.871-1.083)	0.601
**Viral load ($\log_{10}$ copies/mL)**					
Mean±SD	4.07±1.44	5.29±1.49	3.80±1.30	2.544 (1.429-4.528)	0.002
**Underlying disease [no. (%)]**					
Hypertension	51 (26.0)	9 (25.7)	42 (26.1)	1.265 (0.481-3.327)	0.634
Diabetes	17 (8.7)	4 (11.4)	13 (8.1)	1.391 (0.339-5.714)	0.647
Hepatitis	1 (0.5)	0 (0.0)	1 (0.6)		1.000
Heart disease	16 (8.2)	2 (5.7)	14 (8.7)	0.804 (0.162-3.994)	0.790
Cerebrovascular disease	7 (3.6)	3 (8.6)	4 (2.5)	4.424 (0.789-24.810)	0.091
Chronic obstructive pulmonary disease	7 (3.6)	4 (11.4)	3 (1.9)	5.628 (0.954-33.184)	0.056
Gastrointestinal Diseases	6 (3.1)	1 (2.9)	5 (3.1)	1.611 (0.159-16.374)	0.687
**Laboratory index (Mean±SD)**					
*WBC ($\times 10^9$/L)	4.23±3.82	5.34±6.04	3.98±3.11	1.077 (0.986-1.177)	0.100
*PLT ($\times 10^9$/L)	48.29±26.26	37.66±26.94	50.61±25.61	0.978 (0.959-0.998)	0.034
*EO% (%)	0.12±0.33	0.04±0.12	0.14±0.35	0.010 (0.000-2.563)	0.104
RDW (%)	13.61±0.97	13.96±0.89	13.54±0.97	2.440 (1.392-4.278)	0.002
*PT (secs)	11.69±2.13	12.49±2.63	11.52±1.98	1.199 (1.005-1.429)	0.043
*APTT (secs)	43.76±14.27	54.30±18.10	41.60±12.35	1.057 (1.025-1.090)	<0.001
TT (secs)	21.94±15.32	24.23±12.34	21.46±15.87	1.010 (0.988-1.0320)	0.368
*DD (ng/ml)	1571.38±1990.26	2372.79±3064.05	1392.05±1620.83	1.000 (1.000-1.000)	0.016
*CK (U/L)	1097.90±2018.17	2219.56±2039.05	898.66±1954.61	1.000 (1.000-1.000)	0.082
*CKMB (U/L)	47.84±60.26	102.35±102.85	37.36±40.87	1.012 (1.005-1.020)	0.001
*LDH (U/L)	915.99±725.94	1695.11±1055.50	783.99±559.57	1.002 (1.001-1.003)	<0.001
*AST (U/L)	372.62±501.13	645.29±570.40	313.34±465.97	1.001 (1.000-1.003)	0.002
*AST/ALT	3.05±1.95	4.66±2.92	2.72±1.48	1.597 (1.249-2.042)	<0.001
*DBIL (μmol/L)	8.58±14.28	15.55±29.05	7.05±7.47	1.035 (1.002-1.069)	0.040
TP (g/L)	56.76±6.91	54.19±8.06	57.32±6.53	0.939 (0.876-1.007)	0.078
ALB (g/L)	29.64±3.83	27.87±3.70	30.04±3.76	0.8242 (0.749-0.946)	0.004
GGT (U/L)	106.10±135.19	174.81±153.98	91.54±126.71	1.004 (1.001-1.007)	0.004
*ALP (U/L)	105.24±80.87	149.50±102.97	95.85±72.37	1.006 (1.002-1.011)	0.005
*TBA (μmol/L)	13.42±29.62	32.97±61.88	9.04±11.61	1.031 (1.005-1.058)	0.021
BUN (mmol/L)	9.89±5.44	8.37±4.04	8.02±3.60	1.034 (0.990-1.080)	0.134
*CREA (μmol/L)	95.66±87.79	148.06±139.84	84.20±66.98	1.007 (1.002-1.012)	0.010
*UA (μmol/L)	288.77±157.41	408.44±237.97	262.84±119.89	1.006 (1.003-1.009)	<0.001
*CO2 (mmol/L)	21.01±4.93	17.82±5.75	21.74±4.44	0.838 (0.761-0.923)	<0.001
*PCT (ng/ml)	2.64±16.01	9.01±34.86	1.15±5.19	1.059 (0.997-1.125)	0.062
*IL-6 (pg/mL)	220.06±664.85	841.84±1358.41	77.93±162.25	1.003 (1.001-1.005)	0.004
*CRP (mg/L)	20.71±41.40	48.61±92.40	16.44±24.65	1.014 (1.000-1.027)	0.048
SAA (mg/L)	127.45±101.18	223.26±96.00	108.75±91.57	1.011 (1.004-1.019)	0.002
HDL (mmol/L)	0.67±0.32	0.40±0.22	0.72±0.32	0.001 (0.000-0.051)	<0.001
*HSTNI (pg/mL)	1002.09±5419.05	2096.12±8926.06	726.36±4105.50	1.000 (1.000-1.000)	0.305
**Clinical manifestation**					
**Non-specific manifestations**					
Fever	157 (98.7)	30 (96.8)	127 (99.2)	0.238 (0.014-4.063)	0.322

Dizzy	46 (28.9)	5 (16.1)	41 (32.0)	0.409 (0.144-1.157)	0.092
Chills	25 (15.7)	3 (9.7)	22 (17.2)	0.526 (0.145-1.917)	0.331
Headache	37 (23.3)	8 (25.8)	29 (22.7)	1.396 (0.549-3.548)	0.483
Myalgias	26 (16.4)	4 (12.9)	22 (17.2）	0.755 (0.238-2.402)	0.635
Lymphadenopathy	56 (35.2)	15 (48.7)	41 (32.0)	2.273 (0.993-5.204)	0.052
Thrombocytopenia	158 (99.4)	31 (100.0)	127 (99.2)		1.000
Leukocytopenia	148 (93.1)	28 (90.3)	120 (93.8)	0.602 (0.148-2.448)	0.479
Fatigue	109 (68.6)	18 (58.1)	91 (71.1)	0.641 (0.276-1.490)	0.302
Arthralgia	6 (3.8)	0 (0.0)	6 (4.7)		0.999
Chest distress	21 (13.2)	3 (9.7)	18 (14.1)	0.689 (0.186-2.547)	0.576
Multiorgan failure	41 (25.8)	21 (67.7)	20 (15.6)	10.897 (4.350-27.294)	<0.001
Disseminated intravascular coagulation	1 (0.6)	0 (0.0)	1 (0.8)		1.000
**Gastrointestinal symptoms**					
Nausea	66 (41.5)	12 (38.7)	54 (42.2)	1.146 (0.480-2.735)	0.759
Diarrhea	97 (61.0)	21 (67.7)	76 (59.4)	1.665 (0.696-3.983)	0.252
Abdominal pain	41 (25.8)	8 (25.8)	33 (25.8)	0.912 (0.353-2.355)	0.849
Vomiting	70 (44.0)	11(35.5)	59 (46.1)	0.827 (0.335-2.040)	0.679
Poor appetite	81 (50.9)	17 (54.8)	64 (50.0)	1.434 (0.632-3.254)	0.389
**Respiratory symptoms**					
Cough	39 (24.5)	9 (29.0)	30 (23.4)	1.125 (0.437-2.894)	0.808
Sputum	25 (15.7)	5 (16.1)	20 (15.6)	0.766 (0.234-2.504)	0.659
Dyspnea	38 (23.9)	23 (74.2)	15 (11.7)	20.612 (7.722-55.022)	<0.001
**Haemorrhagic symptoms**					
Ecchymosis	15 (9.4)	4 (12.9)	11 (8.6)	1.864 (0.536-6.490)	0.328
Macroscopic hematuria	3 (1.9)	2 (6.5)	1 (0.8)	9.472 (0.809-110.947)	0.073
Tract bleeding	23 (14.5)	10 (32.3)	13 (10.2)	3.874 (1.427-10.514)	0.008
**Neurological symptoms**					
Drowsiness	36 (22.6)	14 (45.2)	22 (17.2)	4.180 (1.691-10.332)	0.002
Neural trance	29 (18.2)	15 (48.4)	14 (10.9)	6.701 (2.679-16.762)	<0.001
Conscious disturbance	25 (15.7)	15 (48.4)	10 (7.8）	11.879 (4.315-32.703)	<0.001

Statistical analyses of categorical variables including sex, underlying diseases and clinical manifestations were performed using χ^2 test and Fisher's exact test. Statistical analyses of continuous variables including age, days since symptom onset, viral load and laboratory index were performed using Chi-square test, one-way ANOVA. "*" in laboratory index used and non-parameter Kruskal-Wallis test.
