# Supplementary material for: Extensive genetic diversity of severe fever with thrombocytopenia syndrome virus circulating in Hubei Province, China, 2018–2022
Source: PLoS Negl Trop Dis. 2023 Sep 18;17(9):e0011654. doi: 10.1371/journal.pntd.0011654 (PMC10538666; doi:10.1371/journal.pntd.0011654)
Supplement: S5 Table — (PDF) [file pntd.0011654.s005.pdf]

S5 Table. Confirmation of the SFTSV segment reassortment by seven methods using RDP packages.

| Recombinant sequence | Minor parental sequence | Major parental sequence | Breakpoint positions | | Detection Methods | | | | | | |
|---|---|---|---|---|---|---|---|---|---|---|---|
| | | | Begin | End | RDP | GENECONV | Bootscan | Maxchi | Chimaera | SiSscan | 3Seq |
| HBHG2021-04 /Huanggang_Wuxue/2021 (C2/C2/C3) | HBHG2020-12 /Huanggang_Hongan/2020 (C3/C3/C3) | BX-2010 /Henan/2010 (C2/C2/C2) | 9808 | 11372 | ** | NS | * | * | * | NS | NS |
| HBHG2022-36 /Huanggang_Luotian/2022 (J3/C3/C3) | HB153/Hubei/2012 (C3/C3/C3) | HB2017-17 /Huanggang_Luotian/2017 (J3/J3/J3) | 6446 | 11482 | *** | *** | *** | *** | *** | *** | *** |
| HBSZ2022-54 /Suizhou/2022 (C3/C2/C3) | HB153/Hubei/2012 (C3/C3/C3) | BX-2010 /Henan/2010 (C2/C2/C2) | 6396 | 9740 | *** | *** | *** | *** | *** | *** | *** |

***p<10-20; **p<10-10; *p<10-3; NS=not significant (p>0.05). Minor parent, parent contributing the smaller fraction of sequence. Major parent, parent contributing the larger fraction of sequence.
