# Supplementary material for: Extensive genetic diversity of severe fever with thrombocytopenia syndrome virus circulating in Hubei Province, China, 2018–2022
Source: PLoS Negl Trop Dis. 2023 Sep 18;17(9):e0011654. doi: 10.1371/journal.pntd.0011654 (PMC10538666; doi:10.1371/journal.pntd.0011654)
Supplement: S6 Table — (PDF) [file pntd.0011654.s006.pdf]

S6 Table. SFTSV recombination events detected using the RDP package.

Segment	Recombinant Segment	Major Parent	Minor Parent	RDPRC	Tools
L	HBSZ2022-23 /Suizhou/2022 (C3)	2011YPQ11 /Henan_Xinyang/2011 (C3)	HBSZ2022-29 /Suizhou/2022 (C2)	0.751	RDP, GENECONV, MaxChi, Chimaera, Siscan, 3Seq
M	HBXG2020-26 /Xiaogan_Dawu/2020 (C4)	2010-FQM /Henan/2011 (C4)	HBXG2022-07 /Xiaogan_Xiaochang /2022 (C3)	0.660	RDP, GENECONV, Bootscan, MaxChi, Chimaera, Siscan, 3Seq
	HBSZ2022-11 /Suizhou/2022 (C2)	2011YPQ11 /Xinyang/2011 (C3)	HB2016-106 /Shiyan/2016 (C2)	0.744	RDP, GENECONV, MaxChi, Chimaera, Siscan, 3Seq
	HBHG2022-12 /Huanggang_Qichun /2022 (J3)	HBHG2021-02 /Huanggang_Luotian /2021 (J3)	SDLZtick12 /China/2010 (C2)	0.762	RDP, GENECONV, Bootscan, MaxChi, Chimaera, Siscan, 3Seq
S	HBXG2022-8 /Xiaogan_Xiaochang /2022 (C3)	HB2016-038 /Huanggang_Yingshan/2016 (C1)	HBSZ2022-30 /Suizhou/2022 (C3)	0.483	RDP, GENECONV, MaxChi, Chimaera, Siscan, 3Seq
	HBSZ2022-27 /Suizhou/2022 (C2)	HNXY_212 /Henan/2013 (C3)	HBSZ2021-28 /Suizhou/2021 (C2)	0.744	RDP, GENECONV, MaxChi, Chimaera, Siscan, 3Seq
	HBXG2022-09 /Xiaogan_Dawu/2022 (C2)	2010-FQM /Henan/2011 (C4)	HB2016-013 /Xiaogan/2016 (C2)	0.736	GENECONV, MaxChi, Chimaera, Siscan, 3Seq
	HBSZ2022-11 /Suizhou/2022 (C2)	HNXY_212 /Henan/2013 (C3)	HB2016-106 /Shiyan/2016 (C2)	0.744	RDP, GENECONV, MaxChi, Chimaera, Siscan, 3Seq
