# Supplementary material for: Extensive genetic diversity of severe fever with thrombocytopenia syndrome virus circulating in Hubei Province, China, 2018–2022
Source: PLoS Negl Trop Dis. 2023 Sep 18;17(9):e0011654. doi: 10.1371/journal.pntd.0011654 (PMC10538666; doi:10.1371/journal.pntd.0011654)
Supplement: S7 Table — (PDF) [file pntd.0011654.s007.pdf]

S7 Table. Logistic regression analysis of variables associated with mortality.

	Univariate OR (95% CI)	*p* value	Multivariate OR (95% CI)	*p* value
Age	1.09 (1.01-1.18)	**0.034**	1.02 (0.93-1.13)	0.639
Sex (M vs. F)	3.00 (0.93-10.76)	0.074	6.58 (1.40-42.59)	**0.027**
Comorbidities (with vs. without)	3.00 (0.93-10.76)	0.074	7.77 (1.54-55.79)	**0.022**
Genotype (C1 vs. C2)	6.00 (0.80-56.33)	0.085	29.68 (2.06-619.27)	**0.018**
Genotype (C3 vs. C2)	0.63 (0.12-2.94)	0.564	0.53 (0.07-3.16)	0.496
Genotype (J3 vs. C2)	0.71 (0.13-3.31)	0.664	0.58 (0.08-3.40)	0.551

Definition of abbreviations: CI = confidence interval: OR = odds ratio: M=Male; F=Female. Genotype C2 exhibits the largest sample size, it was chosen for the comparison. Genotype C4 was deleted because there was no dead case in it. *p* values below 0.05 are in bold.
