# Supplementary material for: Extensive genetic diversity of severe fever with thrombocytopenia syndrome virus circulating in Hubei Province, China, 2018–2022
Source: PLoS Negl Trop Dis. 2023 Sep 18;17(9):e0011654. doi: 10.1371/journal.pntd.0011654 (PMC10538666; doi:10.1371/journal.pntd.0011654)
Supplement: S8 Table — (PDF) [file pntd.0011654.s008.pdf]

S8 Table. Laboratory index and presence of severe clinical manifestations during hospitalization among SFTS patients infected with different viral genotypes.

	**Genotype C1 (n=5)**	**Genotype C2 (n=25)**	**Genotype C3 (n=21)**	**Genotype C4 (n=6)**	**Genotype J3 (n=20)**	***p* value**
**Underlying disease [no. (%)]**						
Hypertension	0 (0.0)	7/25 (28.0)	3/21 (14.3)	1/6 (16.7)	9/20 (45.0)	0.103
Diabetes	0 (0.0)	1/25 (4.0)	1/21 (4.8)	2/6 (33.3)	3/20 (15.0)	0.178
Heart disease	0/5 (0.0)	3/25 (12.0)	1/21 (4.8)	0/6 (0.0)	0/20 (0.0)	0.582
Cerebrovascular disease	0/5 (0.0)	0/25 (0.0)	1/21 (4.8)	0/6 (0.0)	1/20 (5.0)	0.650
Chronic obstructive pulmonary disease	1/5 (20.0)	0/25 (0.0)	1/21 (4.8)	0/6 (0.0)	0/20 (0.0)	0.089
Gastrointestinal diseases	0/5 (0.0)	1/25 (4.0)	1/21 (4.8)	1/6 (16.7)	0/20 (0.0)	0.440
**Case fatality rate (CFR)**						
No. (%)	3/5 (60.0)	6/26 (23.1)	3/22 (13.6)	0/6 (0.0)	3/20 (15.0)	0.147
**Clinical manifestation**						
Multiorgan failure	2/2 (100.0)	3/13 (23.1)	3/16 (18.8)	1/6 (16.7)	5/15 (33.3)	0.223
Dyspnea	2/2 (100.0)	3/13 (23.1)	2/16 (12.5)	1/6 (16.7)	5/15 (33.3)	0.125
Tract bleeding	1/2 (50.0)	3/13 (23.1)	3/16 (18.8)	0/6 (0.0)	4/15 (26.7)	0.542
Drowsiness	1/2 (50.0)	5/13 (38.5)	3/16 (18.8)	0/6 (0.0)	8/15 (53.3)	0.082
Neural trance	1/2 (50.0)	3/13 (23.1)	2/16 (12.5)	1/6 (16.7)	7/15 (46.7)	0.201
Conscious disturbance	2/2 (100.0)	0/13 (0.0)	2/16 (12.5)	1/6 (16.7)	6/15 (40.0)	0.005
**Viral load(log10copies/mL)**						
Mean±SD	3.96±0.13	4.20±1.63	3.95±1.48	3.76±0.67	4.03±0.94	0.982
**Laboratory index (Mean±SD)**						
PLT ($\times 10^9$/L)	40.00±8.80	43.13±19.19	45.82±21.81	44.50±29.14	53.25±20.16	0.511
APTT (secs)	47.55±6.47	50.66±21.45	44.94±14.70	38.86±8.62	40.91±9.70	0.281
DD (ng/ml)	1286.32±893.97	1386.36±1495.01	1021.94±963.97	768.20±440.24	1093.59±1165.86	0.800
CKMB (U/L)	30.09±34.79	39.45±47.54	32.71±26.61	37.40±19.78	64.07±67.36	0.263
LDH (U/L)	919.60±312.43	1175.79±1008.27	785.37±397.52	601.60±239.75	1004.89±552.64	0.306
AST (U/L)	412.80±206.60	502.44±628.39	309.32±361.79	199.00±100.18	359.65±291.57	0.468
AST/ALT	3.55±1.13	3.57±2.46	3.28±2.65	2.68±0.94	3.22±1.53	0.934
DBIL (μmol/L)	3.85±1.97	7.38±8.57	4.69±2.50	2.87±1.52	13.86±9.97	0.002
ALB (g/L)	31.38±2.74	29.85±4.96	30.05±3.54	27.90±5.29	29.38±4.24	0.755
GGT (U/L)	67.33±17.39	101.59±125.81	73.95±106.52	46.33±33.45	221.36±239.56	0.037
ALP (U/L)	167.33±91.27	91.59±45.63	90.41±68.60	72.67±19.93	181.79±157.08	0.014
TBA (μmol/L)	5.30	29.01±69.97	11.34±17.33	6.35±8.62	17.19±15.11	0.699
CREA (μmol/L)	101.86±41.63	87.62±48.01	84.98±38.49	78.27±37.32	83.82±50.07	0.925
UA (μmol/L)	365.58±177.44	276.72±129.51	243.09±80.25	210.05±33.10	273.69±151.13	0.275
CO2 (mmol/L)	15.80	21.74±5.59	22.43±4.29	26.08±4.38	19.78±4.93	0.080
IL-6 (pg/mL)	110.05±64.76	195.85±252.41	109.36±268.28	307.28±579.85	159.62±430.07	0.857
CRP (mg/L)	6.16±2.91	13.51±14.11	16.97±21.02	6.63±4.92	25.38±62.75	0.893
SAA (mg/L)	346.24±91.59	130.17±81.79	70.41±64.03	60.53±51.87	125.42±110.88	0.003
HDL (mmol/L)	0.27	0.68±0.20	0.54±0.22	0.70±0.26	0.66±0.37	0.522

P value: comparison of laboratory index and presence of severe clinical manifestations among five viral genotypes by χ^2 test, Fisher's exact test or One-Way ANOVA test
