# Supplementary material for: Extensive genetic diversity of severe fever with thrombocytopenia syndrome virus circulating in Hubei Province, China, 2018–2022
Source: PLoS Negl Trop Dis. 2023 Sep 18;17(9):e0011654. doi: 10.1371/journal.pntd.0011654 (PMC10538666; doi:10.1371/journal.pntd.0011654)
Supplement: S9 Table — (PDF) [file pntd.0011654.s009.pdf]

S9 Table. Comparison of mortality-associated laboratory variables between patients infected with Genotype C1 and each of other four viral genotypes by One-Way ANOVA test.

	**SAA (mg/L)**
Genotype C2	0.002
Genotype C3	<0.001
Genotype C4	<0.001
Genotype J3	<0.001
