# Supplementary material for: Extensive genetic diversity of severe fever with thrombocytopenia syndrome virus circulating in Hubei Province, China, 2018–2022
Source: PLoS Negl Trop Dis. 2023 Sep 18;17(9):e0011654. doi: 10.1371/journal.pntd.0011654 (PMC10538666; doi:10.1371/journal.pntd.0011654)
Supplement: S10 Table — (PDF) [file pntd.0011654.s010.pdf]

S10 Table. Acronym list.

Abbreviation	Full name
SFTS	Severe Fever With Thrombocytopenia Syndrome
SFTSV	Severe Fever With Thrombocytopenia Syndrome virus
CFR	Case fatality rate
RdRp	RNA-dependent RNA polymerase
GP	Envelope glycoprotein
NP	Nucleoprotein
NSs	Nonstructural protein
RT-PCR	Reverse transcription polymerase chain reaction
SD	Standard Deviation
IQR	Inter quartile range
ANOVA	Analysis of variance
OR	Odds ratio
CDS	Coding sequence
WBC	White Blood Cell
PLT	Platelet
EO%	Eosinophil %
RDW	Red blood cell distribution width
PT	Prothrombin time
APTT	Activated Partial Thromboplastin Time
TT	Thrombin Time
DD	D-Dimer
CK	Creatine Kinase
CKMB	Creatine K Inase-MB
LDH	Lactate Dehydrogenase
AST	Aspartate Aminotransferase
AST/ALT	Aspartate Aminotransferase/Alanine Transaminase
DBIL	Direct bilirubin
TP	Total protein
ALB	Albumin
GGT	γ-glutamyl Transpeptadase
ALP	Alkaline Phosphatase
TBA	Total bile acid
BUN	Blood urea nitrogen
CREA	Creatinine
UA	Uric Acid
CO2	Carbon dioxide
PCT	Procalcitonin
IL-6	Interleukin-6
CRP	C-reactive protein
SAA	Serum amyloid A
HDL	High density lipoprotein
HSTNI	Hypersensitive troponin
