# Supplementary figures and images for: Extensive genetic diversity of severe fever with thrombocytopenia syndrome virus circulating in Hubei Province, China, 2018–2022
Source: PLoS Negl Trop Dis. 2023 Sep 18;17(9):e0011654. doi: 10.1371/journal.pntd.0011654 (PMC10538666; doi:10.1371/journal.pntd.0011654)

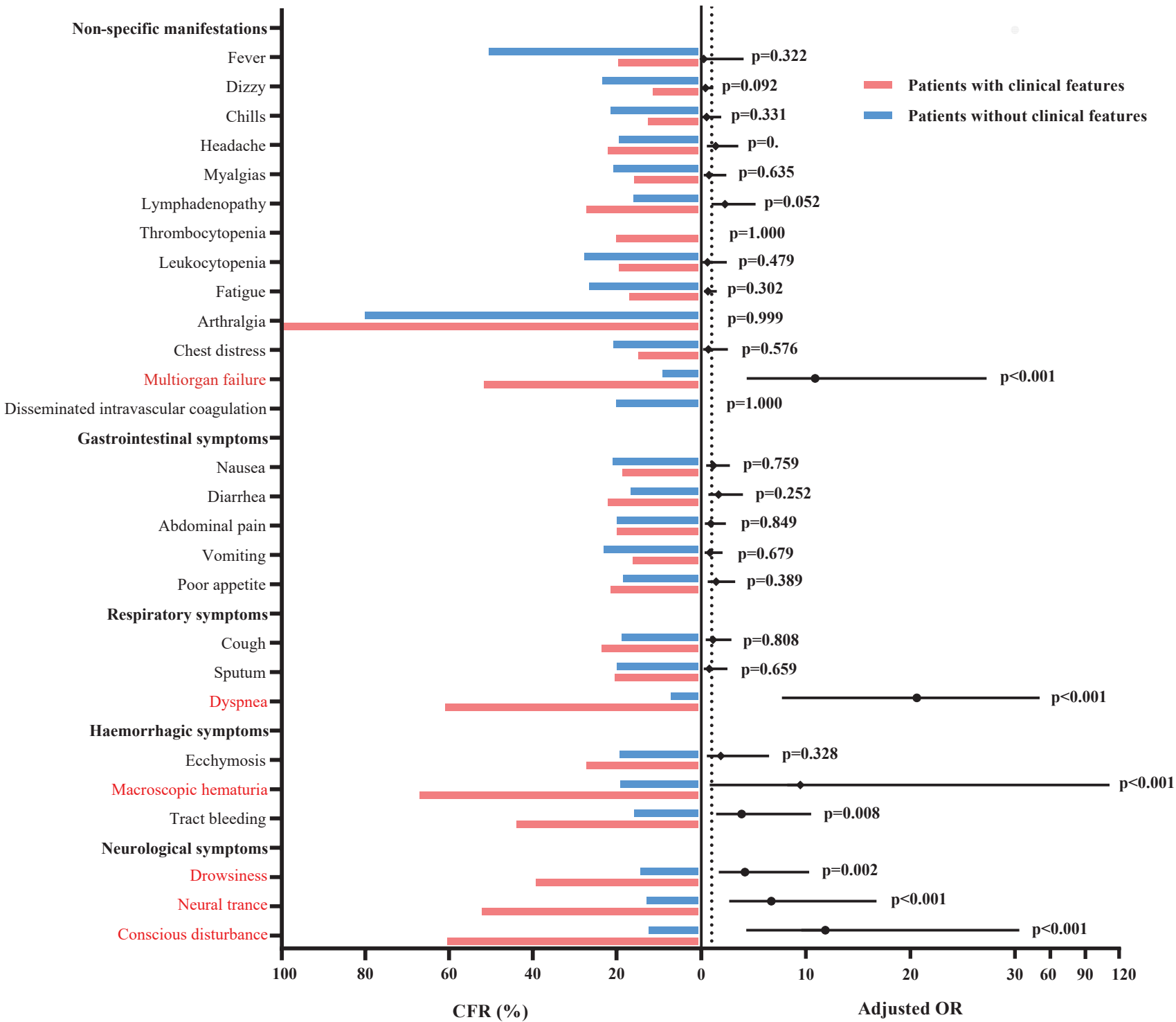

Supplement: S1 Fig — The black points are the adjusted ORs for death and the black error bars are the 95% CIs. ORs were adjusted for age, sex, and delay from symptom onset to admission. The adjusted OR for macroscopic hematuria have a higher scale than the other symptoms. The dotted line indicates an adjusted OR of 1. CFR = case fatality rate. OR = odds ratio. (PDF) [file pntd.0011654.s011.pdf]

China

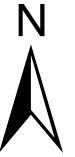

Genotype

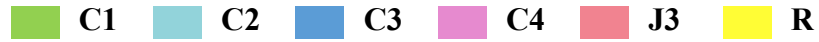

Identified cases:

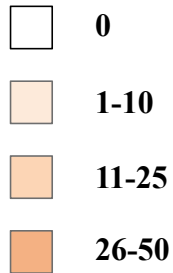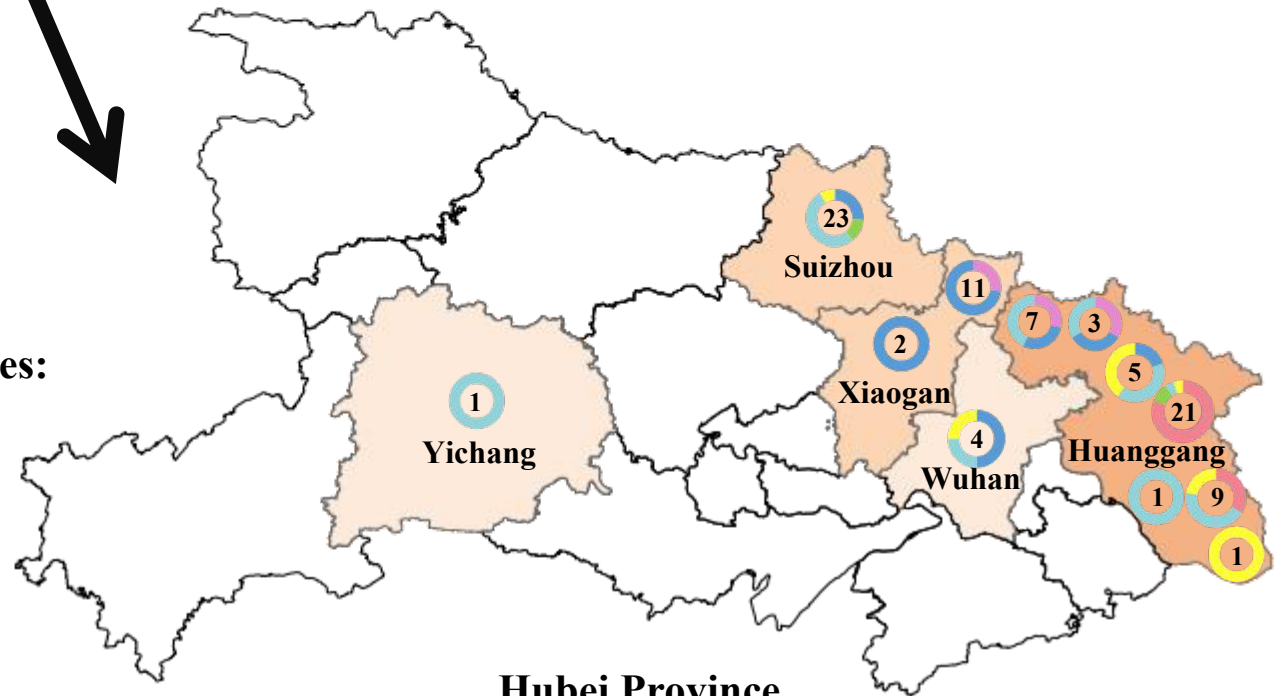

Hubei Province

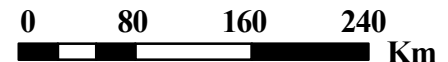

Supplement: S2 Fig — The map was created in ArcGIS 10.2 software (ESRI Inc., Redlands, CA, USA) and modified using Adobe illustrator, Version CC2018 (Adobe, San Jose, CA, USA). The source of the base layer shapefile was from the open access platform: National Platform for Common Geospatial Information Services (www.tianditu.gov.cn). (PDF) [file pntd.0011654.s012.pdf]

A

J3

J2

J1

C6

C5

C4

C2

C1

C3

0.005

B

J3

J2

J1

C5

C4

C2

C1

C3

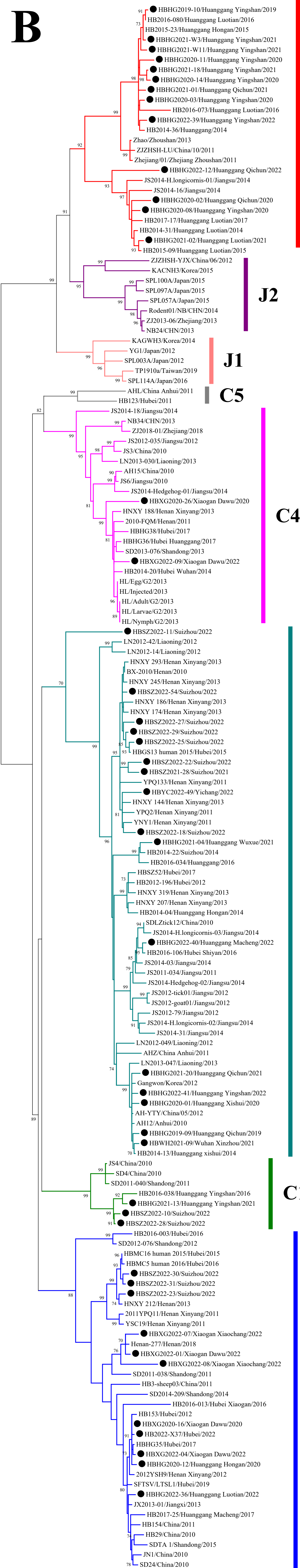

0.005

C

J3

J4

J2

J1

C2

C1

C4

C3

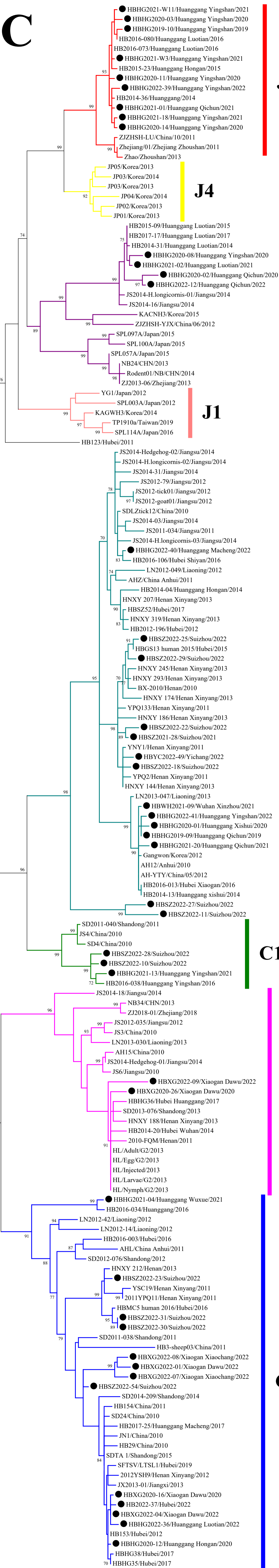

0.005

Supplement: S3 Fig — Phylogenetic analysis based on the complete ORF sequences of (A) L, (B) M, and (C) S segments of Hubei SFTSVs and all available reference sequences. The full version of the Maximum Likelihood (ML) trees based on the Kimura 2-parameter model were constructed and tested by bootstrap analysis with 1,000 replications. The scale bar indicates the number of nucleotide substitutions per site, and the phylogenetic branches were supported with greater than 70% bootstrap values. (PDF) [file pntd.0011654.s013.pdf]
